# Repurposed immunomodulatory drugs for Covid-19 in pre-ICu patients - mulTi-Arm Therapeutic study in pre-ICu patients admitted with Covid-19 – Repurposed Drugs (TACTIC-R): A structured summary of a study protocol for a randomised controlled trial

**DOI:** 10.1186/s13063-020-04535-4

**Published:** 2020-07-08

**Authors:** Spoorthy Kulkarni, Marie Fisk, Michalis Kostapanos, Edward Banham-Hall, Simon Bond, Elena Hernan-Sancho, Sam Norton, Joseph Cheriyan, Andrew Cope, James Galloway, Frances Hall, David Jayne, Ian B. Wilkinson

**Affiliations:** 1 Cambridge University Hospitals NHS Foundation Trust, Cambridge Biomedical Campus, Hills Road, Cambridge, CB2 0QQ UK; 2 King’s College London, Strand, London, WC2R 2LS UK; 3 University of Cambridge, The Old Schools, Trinity Lane, Cambridge, CB2 1TN UK

**Keywords:** COVID-19, Randomised controlled trial, Protocol, Baricitinib, Ravulizumab, Open-label, Adaptive trial, Repurposed drugs

## Abstract

**Objectives:**

To determine if a specific immunomodulatory intervention reduces progression of COVID-19-related disease to organ failure or death, compared to standard of care (SoC).

**Trial design:**

Randomised, parallel 3-arm (1:1:1 ratio), open-label, Phase IV platform trial of immunomodulatory therapies in patients with late stage 1 or stage 2 COVID-19-related disease, with a diagnosis based either on a positive assay or high suspicion of COVID-19 infection by clinical and/or radiological assessment.

**Participants:**

Patients aged 18 and over, with a clinical picture strongly suggestive of COVID-19-related disease (with/without a positive COVID-19 test) AND a Risk count (as defined below) >3 OR ≥3 if risk count includes “Radiographic severity score >3”. A risk count is calculated by the following features on admission (1 point for each): radiographic severity score >3, male gender, non-white ethnicity, diabetes, hypertension, neutrophils >8.0 x10^9^/L, age >40 years and CRP >40 mg/L.

Patients should be considered an appropriate subject for intervention with immunomodulatory therapies in the opinion of the investigator and be able to be maintained on venous thromboembolism prophylaxis during the inpatient dosing period, according to local guidelines. The complete inclusion and exclusion criteria as detailed in the additional file 1 should be fulfilled. Patients will be enrolled prior to the need for invasive mechanical ventilation, cardiac or renal support. Participants will be recruited across multiple centres including initially at Cambridge University Hospitals NHS Foundation Trust, King’s College Hospital NHS Foundation Trust, Guy’s and St Thomas’ NHS Foundation Trust, University Hospital of Wales, Gloucestershire Royal Hospitals NHS Foundation Trust and The Royal Wolverhampton NHS Trust.

**Intervention and comparator:**

Each active comparator arm will be compared against standard of care (SoC). The immunomodulatory drugs were selected from a panel of licenced candidates by a drug evaluation committee, which considered potential efficacy, potential toxicity, scalability and novelty of each strategy. The initial active arms comprise baricitinib and ravulizumab.

Baricitinib will be given 4 mg orally (once daily (OD)) on days 1-14 or until day of discharge. The dose will be reduced to 2 mg OD for patients aged > 75 years and those with an estimated Cockcroft Gault creatinine clearance of 30-60 ml/min.

Ravulizumab will be administered intravenously once according to the licensed weight-based dosing regimen (see Additional file 1).

Each active arm will be compared with standard of care alone. No comparisons will be made between active arms in this platform trial.

**Main outcomes:**

The primary outcome is the incidence (from baseline up to Day 14) of any one of the events (whichever comes first): death, invasive mechanical ventilation, extra corporeal membrane oxygenation, cardiovascular organ support (inotropes or balloon pump), or renal failure (estimated Cockcroft Gault creatinine clearance <15ml/min).

**Randomisation:**

Eligible patients will be randomised using a central web-based randomisation service (Sealed Envelope) in a 1:1:1 ratio, stratified by site to one of the treatment arms or SoC.

**Blinding (masking):**

This is an open-label trial. Data analysis will not be blinded.

**Numbers to be randomised (sample size):**

There is no fixed sample size for this study. Serial interim analyses will be triggered by an Independent Data Monitoring Committee (IDMC), including analysis after 125 patients are recruited to each arm, 375 in total assuming 3 arms. Additional interim analyses are projected after 229 patients per arm, and potentially then after 469 per arm, but additional analyses may be triggered by the IDMC.

**Trial Status:**

TACTIC-R Protocol version number 2.0 date May 20, 2020, recruitment began May 7, 2020 and the end trial will be the date 18 months after the last patient’s last visit. The recruitment end date cannot yet be accurately predicted.

**Trial registration:**

Registered on EU Clinical Trials Register EudraCT Number: 2020-001354-22 Registered: 6 May 2020

It was registered on ClinicalTrials.gov (NCT04390464) and on ISRCTN (ISRCTN11188345)

**Full protocol:**

The full protocol is attached as an additional file, accessible from the Trials website (Additional file 1). In the interest in expediting dissemination of this material, the familiar formatting has been eliminated; this Letter serves as a summary of the key elements of the full protocol.

## Supplementary information

**Additional file 1.** Full Study Protocol.

## Data Availability

Not applicable. Ownership of the data arising from this trial resides with the trial team and the sponsor.

