# Supplementary material for: Repurposed immunomodulatory drugs for Covid-19 in pre-ICu patients - mulTi-Arm Therapeutic study in pre-ICu patients admitted with Covid-19 – Repurposed Drugs (TACTIC-R): A structured summary of a study protocol for a randomised controlled trial
Source: Trials. 2020 Jul 8;21:626. doi: 10.1186/s13063-020-04535-4 (PMC7341462; doi:10.1186/s13063-020-04535-4)
Supplement: Supplementary file 1 — Additional file 1. Full Study Protocol. [file 13063_2020_4535_MOESM1_ESM.pdf]

Clinical Trial Protocol

Trial Title: **mulTi-Arm Therapeutic study in pre-ICu patients admitted with Covid-19 – Repurposed Drugs (TACTIC-R)**

Protocol Number: TACTIC-R

EudraCT Number: 2020-001354-22

ClinicalTrials.gov Number: NCT04390464

ISRCTN Number: ISRCTN11188345

Investigational Product: Baricitinib, Ravulizumab

Protocol Version: v2.0 20MAY2020

Chief Investigator: Dr Frances Hall
CI Address: Cambridge University Hospitals NHS Foundation Trust,
Box 194, Unit E6,
Hills Road,
Cambridge,
CB2 0QQ

Telephone: 01223 274915

Trial Sponsor: Cambridge University Hospitals NHS Foundation Trust

SAE Reporting: TACTIC Clinical Trial Coordinator

Email: Cambs.cardiovascular@nhs.net and cctupharmacovigilance@addenbrookes.nhs.uk

1 Protocol Signatures:

I give my approval for the attached protocol entitled "mulTiArm Therapeutic study in pre-Icu patients Admitted with Covid-19 – Repurposed drugs (TACTIC-R)" dated 20 May 2020

Chief Investigator

Name:

Signature: _______________________________________

Date: ___________________________

Site Signatures

I have read the attached protocol entitled "mulTiArm Therapeutic study in pre-Icu patients Admitted with Covid-19 – Repurposed drugs (TACTIC-R)" dated 20 May 2020 and agree to abide by all provisions set forth therein.

I agree to comply with the conditions and principles of Good Clinical Practice as outlined in the European Clinical Trials Directives 2001/20/EC and 2005/28/EC, the Medicines for Human Use (Clinical Trials) Regulations 2004 (SI 2004/1031) and any subsequent amendments of the clinical trial regulations, the Sponsor's SOPs, and other regulatory requirements as amended.

I agree to ensure that the confidential information contained in this document will not be used for any other purpose other than the evaluation or conduct of the clinical investigation without the prior written consent of the Sponsor

Principal Investigator

Name:

Signature: _______________________________________

Date: ___________________________

2 Trial Management Committee(s) and Protocol Contributors

Chief Investigator:	
Dr Frances Hall (CI)	Consultant Rheumatologist, Cambridge University Hospitals NHS Foundation Trust Rheumatology Research Unit, Unit E6, Box 194, Hills Road, Cambridge CB2 0QQ<br><br>Telephone: 01223 274915
Prof David Jayne (Co-Lead)	Director of the Vasculitis and Lupus Service and Professor of Clinical Autoimmunity, Division of Experimental Medicine and Immunotherapeutics, University of Cambridge, Hills Road, Cambridge, CB2 0QQ
Sub-Investigators / Protocol Contributors:	
Dr Joseph Cheriyan	Consultant in Clinical Pharmacology, Directorate of Acute Medicine Services, Division C, Cambridge University Hospitals NHS Foundation Trust and Division of Experimental Medicine & Immunotherapeutics, Dept of Medicine, Univ. of Cambridge, Box 98, Hills Road, Cambridge, CB2 0QQ
Prof Ian Wilkinson	Head of Division, Division of Experimental Medicine & Immunotherapeutics, University of Cambridge & Director, Cambridge Clinical Trials Unit, Cambridge University Hospitals NHS Foundation Trust, Box 98, Hills Road, Cambridge, CB2 0QQ
Prof Andrew Cope	Versus Arthritis Professor of Rheumatology<br>Head, Centre for Rheumatic Diseases<br>Faculty of Life Sciences and Medicine<br>Guy's Campus<br>King's College London<br>London SE1 1UL
Dr James Galloway	Senior Lecturer and Honorary Consultant Rheumatologist<br>Centre for Rheumatic Diseases<br>10 Cutcombe Road,<br>King's College London,<br>London. SE5 9RJ
Dr Michalis Kostapanos	Cambridge University Hospitals NHS Foundation Trust and Division of Experimental Medicine & Immunotherapeutics, Dept. of Medicine, Univ. of Cambridge, Box 98, Hills Road,<br>Cambridge, CB2 0QQ
Dr Edward Banham-Hall	Clinical Trials Physician, Consultant in Acute Medicine, Directorate of Acute Medical Services, Division C, Cambridge University Hospitals NHS Foundation Trust

Dr Helen Parfrey	Consultant Respiratory Physician<br>Cambridge Interstitial Lung Disease Unit, Royal Papworth Hospital NHS Foundation Trust, Parworth Road, Cambridge Biomedical Campus,<br>Cambridge CB2 0AY
Trial coordination:	
Elena Hernan Sancho	Cardiovascular Trials Office, Cambridge Clinical Trials Unit, Box 401, Cambridge University Hospitals NHS Foundation Trust, Hills Road, Cambridge, CB2 0QQ<br>Email: elena.hernansancho@addenbrookes.nhs.uk

Trial statistician:	
Dr Simon Bond	Cambridge Clinical Trials Unit, Box 401, Cambridge University Hospitals NHS Foundation Trust, Hills Road, Cambridge, CB2 0QQ
Dr Sam Norton	Senior Lecturer in Research Methods & Statistics<br>Centre for Rheumatic Diseases<br>10 Cutcombe Road,<br>King's College London,<br>London. SE5 9RJ
Trial pharmacist:	
Dr Lynne Whitehead	Central Pharmacy Level 1, Box 55<br>Cambridge University Hospitals NHS Foundation Trust, Hills Road, Cambridge, CB2 0QQ
Sponsor:	
Cambridge University Hospitals NHS Foundation Trust	Research & Development Department (Box 277)<br>Addenbrooke's Hospital, Hills Road<br>Cambridge, CB2 0QQ<br>Telephone: 01223 245151 e-mail: ccturegulatory@addenbrookes.nhs.uk

Table of Contents

1 Protocol Signatures: 2
2 Trial Management Committee(s) and Protocol Contributors 3
3 Abbreviations 8
4 Trial Flow Chart 10
5 Introduction 11
5.1 Background 11
5.2 Selecting strategies for a therapeutic trial in COVID19 15
5.3 Choice of individual drugs 16
5.3.1 Immune modulation selection 16
5.4 Patient selection for the study 17
5.5 Study proposal 19
5.6 Trial Hypotheses 20
6 Information on Selected Treatments 21
6.1 Baricitinib 21
6.1.1 Mechanism of Action 21
6.1.2 Rationale for use as Therapeutic in COVID-19-related disease 21
6.2 Ravulizumab 23
6.2.1 Mechanism of Action 23
6.2.2 Rationale for use as Therapeutic in COVID-19-related disease 23
7 Trial Design 24
7.1 Statement of Design 24
7.1.1 Addition of treatment arms 24
7.1.2 Termination of treatment arms 24
7.2 Participants Trial Duration 25
7.3 Trial Objectives 25
7.3.1 Primary objective 25
7.3.2 Secondary objectives 25
7.3.3 Exploratory objectives 25
7.4 Trial Outcome Measures 26
7.4.1 Primary outcome measure 26
7.4.2 Secondary outcome measures 26
7.4.3 Exploratory outcome measures 26
8 Selection and withdrawal of participants 26
8.1 Inclusion Criteria 26
8.2 Exclusion Criteria 27
8.3 Treatment Assignment and Randomisation Number 27
8.4 Treatment Cessation Criteria 27
8.5 Consent withdrawal 28
9 Trial Treatments 28
9.1 Treatment Summary 28
9.1.1 Baricitinib 28
9.1.2 Ravulizumab 30
9.2 Active comparator products 31
9.3 Accountability and dispensing 31
9.3.1 Dispensing 31
9.3.2 Drug accountability 32
9.3.3 Returns and destruction 32
10 Procedures and assessments 32

10.1 Participant identification 32
10.2 Consent 32
10.3 Screening evaluation 33
10.3.1 Screening Assessments (Day -2 to Day -1) 33
10.3.2 Participant Randomisation 34
10.4 Baseline Assessments (Day -2 to Day -1) 34
10.5 Trial assessments 35
10.5.1 Timing of in hospital assessments 35
10.5.2 Further assessments and end of trial visit 35
10.6 Schedule of Assessments 36
10.7 End of Trial Participation 38
10.8 Trial restrictions 38
11 Assessment of Safety 38
11.1 Definitions 38
11.1.1 Adverse event (AE) 38
11.1.2 Adverse reaction to an investigational medicinal product (AR) 39
11.1.3 Unexpected adverse reaction 39
11.1.4 Serious adverse event or serious adverse reaction (SAE / SAR) 39
11.1.5 Suspected Unexpected Serious Adverse Reaction (SUSAR) 39
11.1.6 Reference Safety Information (RSI) 39
11.2 Expected Adverse Reactions/Serious Adverse Reactions (AR /SARs) 40
11.3 Expected Adverse Events/Serious Adverse Events (AE/SAE) 40
11.4 Evaluation of adverse events 40
11.4.1 Assessment of seriousness 40
11.4.2 Assessment of causality 41
11.4.3 Clinical assessment of severity 41
11.4.4 Recording of adverse events 41
11.5 Recording and Reporting SAEs and SARs 42
11.6 Reporting of Suspected Unexpected Serious Adverse Reactions (SUSARs) 42
11.6.1 Who should report and whom to report to? 42
11.6.2 When to report? 43
11.6.3 How to report? 43
11.7 Pregnancy Reporting 44
11.8 Adverse events of special interest (AESIs) 44
12 Toxicity – Emergency Procedures 44
13 Evaluation of Results (Definitions and response/evaluation of outcome measures) 44
13.1 Response criteria 44
14 Storage and Analysis of Samples 44
15 Statistics 44
15.1 Statistical methods 44
15.2 Interim analyses 45
15.3 Number of Participants to be enrolled 46
15.4 Procedure to account for missing or spurious data 47
15.5 Definition of the end of the trial 47
16 Data handling and record keeping 47
16.1 CRF 47
16.2 Source Data 48
16.3 Data Protection & Participant Confidentiality 48
17 Data Monitoring Committee 48
18 Trial Steering Committee 49
19 Ethical & Regulatory considerations 49
19.1 Ethical committee review 49
19.2 Regulatory Compliance 49

19.3 Protocol Amendments 49
19.4 Peer Review 49
19.5 Declaration of Helsinki and Good Clinical Practice 50
19.6 GCP Training 50
20 Sponsorship, Financial and Insurance 50
21 Monitoring, Audit & Inspection 50
22 Protocol Compliance and Breaches of GCP 50
23 Publications policy 51
24 References 51
Safety Reporting Flow Chart 53

3 Abbreviations

Ab	Antibody
ACE	Angiotensin Converting Enzyme
ADCC	Antibody dependent cell-mediated cytotoxicity
AE/AR	Adverse event/Adverse Reaction
AESI	Adverse event of special interest
ALP	Alkaline phosphatase
ALT	Alanine transaminase
ARDS	Acute respiratory distress syndrome
AST	Aspartate transaminase
BD	Twice daily
CA	Competent Authority
CAD	Coronary artery disease
CAPS	Cryopyrin-associated periodic syndrome
CH	Clonal Hematopoiesis
COVID-19	Severe Adult Respiratory Syndrome Coronavirus 2
CRC	COVID-19 related complications
CRF	Case Report Form
CRP	C reactive protein
CTIMP	Clinical Trial of Investigational Medicinal Product
CTL	Cytotoxic T Lymphocyte
CYP	Cytochrome P450
DAD	Diffuse Alveolar Damage
DMARD	Disease-modifying anti-rheumatic drug
DC	Dendritic Cell
DMC	Data Monitoring Committee
DNA/RNA	Deoxyribonucleic acid / Ribonucleic acid
DSUR	Development Safety Update Report
DVT	Deep venous thrombosis
ECMO	Extracorporeal membrane oxygenation
FDA	Food and Drug Administration
G-CSF	Granulocyte-colony stimulating factor
GP	General Practitioner
GCP	Good Clinical Practice
GGT	Gamma glutamyl transpeptidase
GM-CSF	Granulocyte-macrophage colony-stimulating factor
HBsAg	Hepatitis B surface antigen
HCV	Hepatitis C virus
HLH	Haemophagocytic Lymphohistiocytosis
hsTroponin	High sensitivity Troponin
IB	Investigator Brochure
ICF	Informed Consent Form
ICU	Intensive Care Unit
IFN	Interferon
IL	Interleukin

IMP	Investigational Medicinal Product
IUD	Intrauterine Device
JAK	Janus Kinase
KLH	Keyhole limpet haemocyanin
LDH	Lactate Dehydrogenase
LFT	Liver/lung function test
LPS	Lipopolysaccharide
MASP	Mannan-binding lectin serine protease
MedDRA	Medical Dictionary for Regulatory Activities
MERS	Middle East Respiratory Syndrome
MHRA	Medicines and Healthcare products Regulatory Agency
MRC	Medical Research Council
MTX	Methotrexate
NK	Natural Killer cell
NIMP	Non Investigational Medicinal Product
NLR	Neutrophil:Lymphocyte Ratio
NT-proBNP	N-terminal pro B type natriuretic peptide
PBMC	Peripheral Blood Mononuclear Cells
PC	Plasma Cell
PCR	Polymerase Chain Reaction
PDGF	Platelet Derived Growth Factor
PE	Pulmonary embolism
PIS	Participant Information Sheet
po	Per oral
qtPCR	Quantitative Polymerase Chain Reaction
R&D	Research and Development
RA	Regulatory Agency
REC	Research Ethics Committee
RSI	Reference Safety Information
rtPCR	Real Time Polymerase Chain Reaction
SAE/SAR	Serious Adverse Event/Serious Adverse Reaction
SBECD	Betadex Sulfobutyl Ether Sodium
SC	Subcutaneously
sHLH	Secondary Haemophagocytic Lymphohistiocytosis
SoC	Standard of Care
SmPC	Summary of Product Characteristics
STAT	Signal Transducer and Activator of Transcription
SUSAR	Suspected Unexpected Serious Adverse Reaction
TB	Tuberculosis
TGF	Tissue Growth Factor
TMG	Trial Management Group
TNF	Tissue Necrosis Factor
TPM	Trial Procedures Manual
TRC	Translational Research Collaboration
TSC	Trial Steering Committee
VTE	Venous thromboembolism
WHO	World Health Organisation

4 Trial Flow Chart

This trial is a randomised, parallel arm, open-label platform trial

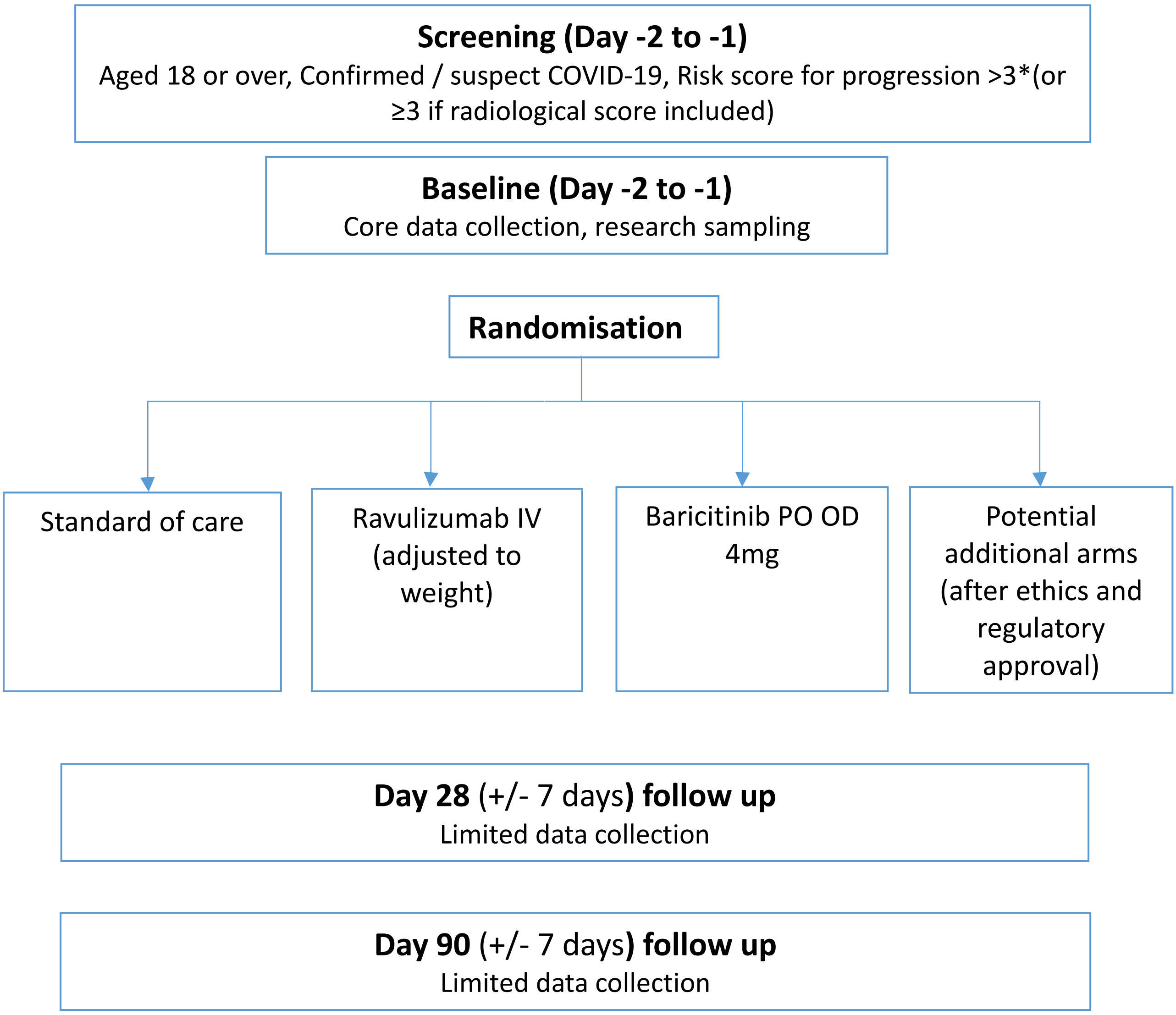

5 Introduction

5.1 Background

The COVID-19 pandemic, as declared on 11th March 2020 by World Health Organisation (WHO), is caused by a novel coronavirus (SARS-Cov-2). In the UK on 17/4/20 108,692 cases had a laboratory-confirmed diagnosis and the total number of COVID-19 associated deaths in hospital totalled 14,576; these data do not cover cases and deaths in the community, where, as yet, no testing is available.

The majority of individuals infected with COVID19 appear to have mild/moderate symptoms, but ~15% have severe disease and there is ~2% mortality across the population. Identified risk factors for severe COVID-19-related disease include age cardiovascular disease, diabetes and male gender. COVID19-related complications (CRC) include acute respiratory distress syndrome, arrhythmia, shock, acute kidney injury, acute cardiac injury, liver dysfunction and secondary infection [Huang et al 2020; King's Critical Care Evidence Summary 09/03/2020]. Currently, there are no current vaccines, prophylactic or therapeutic agents of proven efficacy. The international research community is rapidly accumulating information regarding the pathogenesis of COVID19 infection. The following key themes have emerged to date:

- Early infection is often asymptomatic but these individuals can be infectious.
- A significant proportion of the severe symptoms and life-threatening complications of these viral infections are not driven by primary viral infection but by an excessive host immune response to COVID-19.
- The immune response to COVID19 is more often damaging in older adults and in patients with selected comorbidities
- Severe organ damage in association with COVID19 is accompanied by a dysregulated autoinflammatory and autoimmune syndrome (see Figure 1).

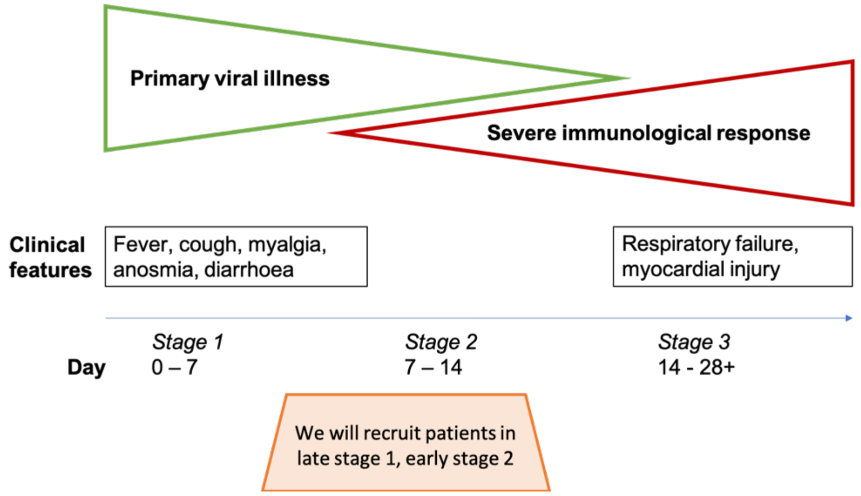

Figure 1: Stages of COVID19

Virology

Coronaviruses are large, enveloped RNA viruses that are distributed widely among mammals and birds. Previous outbreaks of severe respiratory disease caused by coronavirus infections in humans have followed zoonotic transmission. SARS-CoV

was identified in 2003 and MERS-CoV emerged in 2012. COVID-19 or SAR-CoV2 is closely related to SARS-CoV and, both use ACE2 as the receptor for the envelope spike protein of COVID19 and is the means of the virus binding to its target cells. Coronaviruses exhibit a range of strategies for evading the early innate immune response, example by inhibiting the action of type I interferons. This facilitates early viral replication and reduces systemic symptoms in the host.

Cytokine Dysregulation in COVID-19-related Disease (Figure 2)

While SARS-CoV infection evades detection by the immune system in the first 24h of infection, it ultimately induces a massive immune system effector response in the subgroup of people who develop severe COVID19-related disease. This leads to potentially life-threatening lung and sometimes multi-organ damage. It is important to note that the development of Diffuse Alveolar Damage (DAD) is often independent of high-titre viral replication (Peiris et al 2003). The massive immune and inflammatory response in affected lungs includes production of high levels of IL-6, TNF-alpha, IL-1-beta, influx of neutrophils and cytotoxic T cells. A Th2 (IL4, IL13) response from alternatively-activated macrophages, and an associated profibrotic phenotype (including increased TGF-beta and PDGF-alpha production) lead to lung fibrosis. Activation of the coagulation cascade is associated with development of fibrin clots in the alveoli. (Gralinski and Baric 2015).

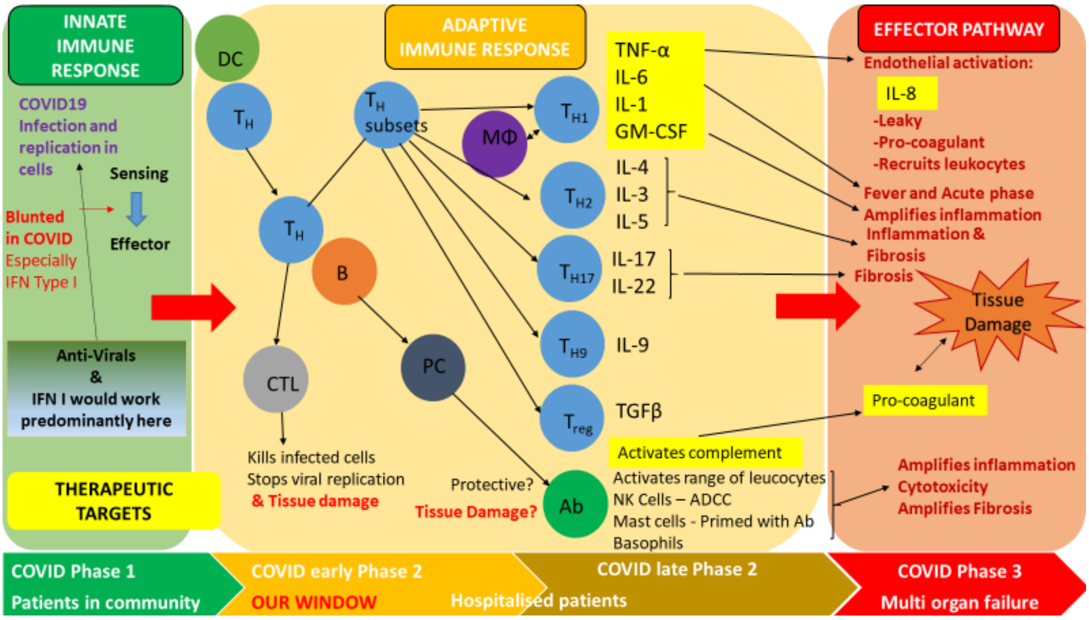

Figure 2. COVID-19 immunopathology and potential therapeutic targets
PC indicates plasma cell, DC-dendritic cell, Ab-antibody, CTL-Cytotoxic T lymphocyte, NK natural killer cells, ADCC-antibody dependent cell-mediated cytotoxicity. TH- T helper cell, IL- interleukin, GM-CSF- Recombinant Human Granulocyte Macrophage Colony-Stimulating Factor

Complement Activation in COVID19-related Disease (Figure 3)

The complement system consists of a series of soluble serine proteases, which circulate in the blood in inactive form. Activation of the complement cascade

results in amplification of the inflammatory response and the formation of a membrane attack complex which perforates cell membranes, thereby causing cell death. The complement cascade can be activated by microbes, including viruses by the alternative pathway. Type I interferon decreases expression of Serping1, a regulator of the complement system and coagulation proteases; this may increase complement-mediated tissue damage and a prothrombotic tendency.

The development of specific antibodies to a target, such as COVID-19 spike protein, focusses complement activation, via the classical pathway, onto membranes expressing the target molecule; this would include infected cells. It would be expected that complement activation, in COVID-19-infected tissues would be markedly amplified after development of COVID-19-specific antibodies, i.e. in stage 2 of the disease. Since both complement components and antibodies circulate in the blood, it is plausible that complement-mediated attack contributes to tissue damage in sites other than the lung, including heart and kidney.

In addition to the anticipated involvement of complement activation in innate and adaptive immune responses to viral infection, COVID-19 infection may activate the cascade by pathogen-specific pathways. There has also been a suggestion that N proteins of SARS-CoV, MERS-CoV and SARS-CoV-2 bind to MASP-2, the key serine protease in the lectin pathway of complement activation (Gao et al 2020).

Direct evidence of complement activation in COVID-19 initially came from research in a mouse model, following the SARS-CoV outbreak in 2002. Mice deficient in C3, the hinge component of the complement system that activates final common pathway, exhibited less weight loss and less respiratory dysfunction. Significantly fewer neutrophils and inflammatory monocytes were present in the lungs of C3 knockout mice than in controls, and subsequent studies revealed reduced lung pathology (Gralinski et al 2018). Recently pulmonary and cutaneous biopsy and autopsy samples from five patients with severe COVID19-related disease have been subjected to immunohistochemistry. This has shown a thrombotic microangiopathy associated with presence of C4d, MASP2 and the membrane attack complex C5b-9 in capillaries (Magro et al 2020).

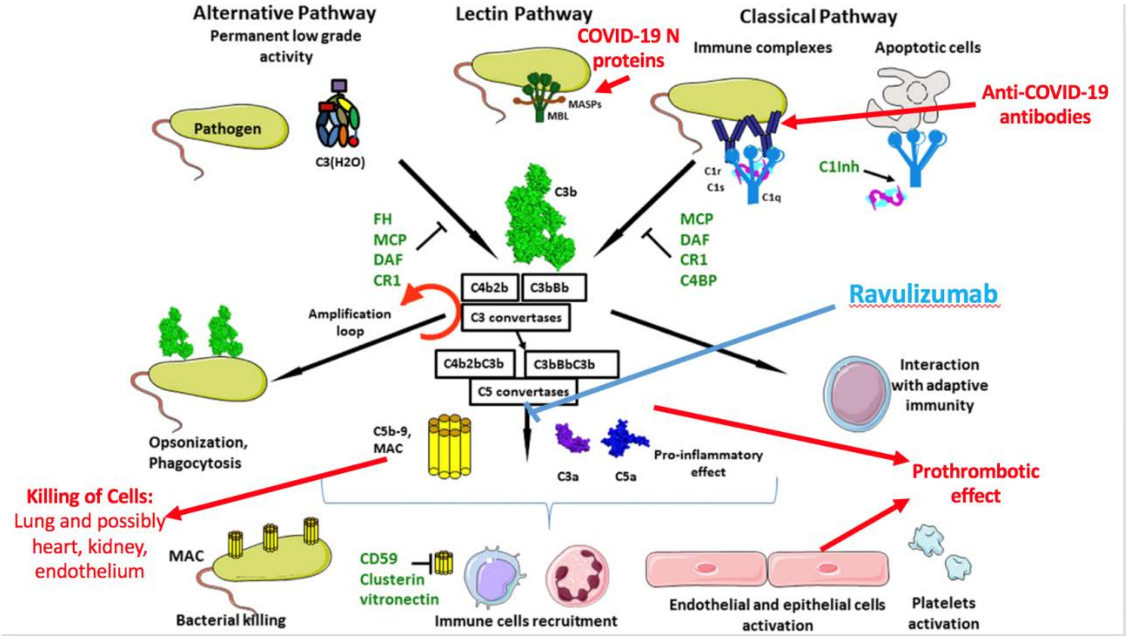

Figure 3 Complement-mediated Pathways in COVID19-related Disease

Blockade of complement activation in stage 2 of COVID-19-related disease may reduce cell killing, inflammatory amplification and activation of the coagulation cascade. Ravulizumab is a monoclonal antibody which prevents activation of complement component C5. This may therefore reduce the thrombotic capillaritis associated with COVID-19-related infection both by decreasing the pro-inflammatory (and neutrophil-recruiting) effect of C5a and C3a and also by decreasing cytolytic formation of the membrane attack complex (MAC).

<u>Precedents for Immunomodulation in Acute Cytokine or Complement Dysregulation Syndromes</u>

There are parallels between COVID-19 ARDS and other dysregulated autoinflammatory syndromes, e.g. secondary haemophagocytic lymphohistiocytosis (sHLH); the latter is most often triggered, in adults, by viral infection (Ramos-Casals et al 2014; Mehta et al 2020). Therapies include immunosuppressives such as cyclophosphamide and ciclosporin A, but several case reports support use of cytokine blockade, e.g. with anakinra or tocilizumab. Paroxysmal nocturnal haemoglobinuria (PNH) is an acquired haemolytic syndrome caused by developing red blood cells which are inadequately protected against complement activation. Treatment with inhibitors of complement C5 activation (eculizumab or ravulizumab) protects patients with PNH against destruction of their red blood cells.

<u>Stratification of Patients with COVID-19-Related Disease</u>

It must be acknowledged that immune modulation is not without risk. Evidence from the field of autoimmune diseases shows that most immune modulation strategies are accompanied by small but significant risks of infection. In the context of COVID19, studies will need to recruit patients who are older, with existing comorbidities (as these are risk factors for COVID19) and with active viral infection. Therefore, it is crucial to select patients in whom the risk of progression to stage 3 disease is sufficient to create clinical equipoise about immune modulatory intervention.

Retrospective data from the COVID19 outbreak in Wuhan, China have revealed divergence of a range of biomarkers, in the early and mid-phases of infection, which discriminate between survivors and non-survivors (Zhou et al 2020); Several of these (D-dimer, neutrophil:lymphocyte Ratio (NLR), ferritin, LDH, diverge early in the clinical course, and IL6 later(Figure 4)).

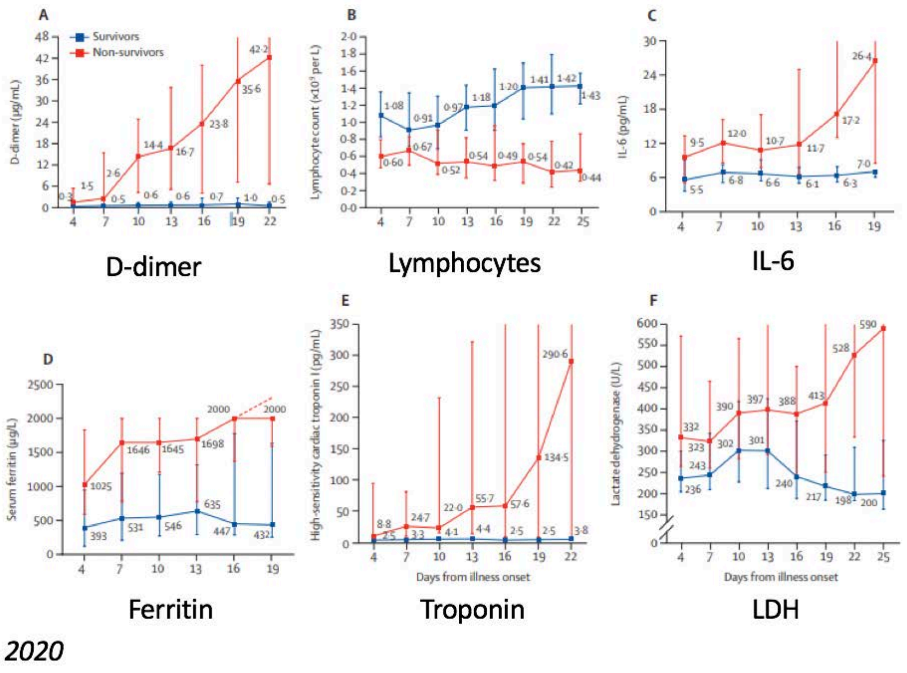

Figure 4: Comparison of Biomarkers in Survivors versus Non-survivors from Wuhan Outbreak of COVID-19 (https://www.researchsquare.com/article/rs-18079/v1)

5.2 Selecting strategies for a therapeutic trial in COVID19

The disease progression supplies important clues to the pathogenesis of the severe immunological phase of the disease (stage 3 in Figure 1). Older adults are preferentially affected (which is unusual for autoimmune diseases). The immunological phase starts consistently around 7-10 days after symptom onset, at the interface of innate and adaptive immune responses.

In addition to these considerations, strategies need to be scalable, with drugs that could, if found to be effective, scaled up for global supply. For these reasons, TACTIC-R is focused on three key paradigms that set the study apart from other contemporary COVID19 studies:

1. **Prompt immune modulatory therapy**
2. **Immune modulation with licensed drugs only with established safety data**
3. **Patient selection for individuals with early (stage 1 / 2) disease but at high risk of complications**

5.3 Choice of individual drugs

5.3.1 Immune modulation selection

There are important clues from the COVID-19 clinical syndrome that influence decisions. First, it is notable that severe immunological diseases are less common in the elderly. In contrast, the severe phase of COVID-19 preferentially affects older persons. This supplies important clues to the immunological drivers and understanding of the changes associated with immune senescence are helpful.

Second, the severe phase of COVID-19 develops predictably around 8-14 days into the illness, a time point at the interface between innate and adaptive immunological responses, and when the viral replication may be on the decline.

Third, published cytokine data through the course of disease show a host inflammatory response that has similarities (e.g. extreme ferritin elevation, IL-6 production, endovascular damage and microvascular thrombotic disease) to known diseases such as haemophagocytic lymphohistiocytosis (HLH) and haemolytic uraemic syndrome (HUS), a disease known to complicate other infections. Furthermore, predictors of fatality from a retrospective, multicentre study of 150 COVID-19 cases from Wuhan, China included typical HLH markers: raised D-dimer, ferritin, LDH, troponin, IL-6 and increased NLR ratio (Zhou et al 2020). This is consistent with organ damage caused by a coronavirus-driven excessive autoinflammatory response.

Several medications licenced for patients with autoimmune disease (rheumatoid arthritis, systemic lupus erythematosus, adult-onset Still's disease) and with HLH are immunomodulatory and may be repurposed to reduce collateral damage caused by the aberrant activation of the immune system in patients with severe COVID-19-related disease (Mehta et al 2020, Richardson et al 2020). Data from the MERS coronavirus outbreak in 2012 provides some evidence for efficacy of this approach, with reports of therapeutic benefit from several immunomodulatory medications (Al-Omari et al 2018) (Gautret et al 2020). However, corticosteroids were unhelpful. Clinical evidence does not support corticosteroid treatment for 2019-nCoV lung injury (Russell et al 2020).

Based upon these data, the TACTIC-R consortium has identified two priority pathways to target:

1. *Cytokine signalling*

Several studies have already launched using cytokine blockage (including a large European anti-IL-6 trial. However, it is important to acknowledge that it is unclear which specific cytokine is responsible for the inflammatory process in COVID-19 (or if the cytokine profiles reflect an epiphenomenon rather than the true pathogenic drive). It is therefore desirable to consider a strategy that offers blockade of more than one cytokine pathway. JAK inhibition offers this potential. Several licensed JAK inhibitors with acceptable safety profiles exist. From the licensed options, Baricitinib is particularly appealing as it selectively targets JAK1/2, blocking IL-6 pathways as well as multiple interferon signalling cascades. Baricitinib is discussed further in section 6.1.

2. *Complement activation*

The complement system is a major mediator of innate immune response. Several mechanisms can trigger activation, including pre-existing antibodies. A theoretical model for COVID19 is that patients with prior exposure to similar but non-SARS-CoV2 coronavirus strains mount a mistaken and ineffective immune response that triggers the severe immunological responses. The time course of the disease would fit with known understanding of complement mediated disease. This theory would also align with older individuals (with a greater antibody repertoire to coronavirus strains) developing disease.

Direct evidence of complement activation in COVID19-related disease is now emerging (see Section 5.1) and this strengthens the rationale for inhibition of the complement cascade. Ravulizumab inhibits activation of complement component C5 and thereby blocks or reduces the development of complement effector functions (see Figure 3).

5.4 Patient selection for the study

Patients will be recruited to TACTIC only if they meet criteria for having a high likelihood of benefit from immunomodulation. We have therefore developed a risk count which identifies patients admitted with a clinical/laboratory diagnosis of COVID19, who are at increased risk of developing severe COVID19-related disease.

Data from the first 200 patients admitted to King's College Hospital (Sneep et al. *under review*) have been used to inform patient selection. Clinical and laboratory data, were modelled using penalised (LASSO) logistic regression to select variables with the most prognostic value. The following variables were considered: Age, gender, non-white ethnicity, radiographic severity on chest radiograph, diabetes, hypertension, neutrophils, lymphocytes, CRP. The radiological severity score was calculated using the method described by Wong et al, 2020. A score of 0-4 was assigned to each lung depending on the extent of involvement by consolidation or ground glass opacities. 0 = no involvement, 1 = <25%, 2 = 25 - 49%, 3= 50 - 75%, 4 = >75% involvement. The scores for each lung were summed to produce a final severity score ranging from 0-8. Radiographs were scored by two emergency department clinicians after a brief training. Interrater reliability was 90.5%.

The outcome modelled was either admission to ICU or death during follow up. The variables selected from the LASSO model were: radiographic severity, male gender, CRP, non-white ethnicity, diabetes, hypertension, and neutrophils (AUC=.86). Age was also selected since It was predictive in a non-linear manner (attributable to a much lower rate of ICU admission with older patients) with an inflection point around 50 years of age. For CRP and Neutrophils the association was linear on the logarithmic scale with no clear threshold effect. For simplicity and pragmatic reasons, cut points were selected for continuous variables at points where risk of poor outcome was consider clinically important. From all the variables, radiographic severity was by far the strongest predictor of progression.

A risk count was calculated by summing (i.e. patients receive 1 point for) each of the following features on admission:

- Radiographic severity score >3
- Male gender
- Non-white ethnicity
- Diabetes
- Hypertension
- Neutrophils >8.0 10^9/L
- Age >40 years
- CRP >40 mg/L

The corresponding risks of ICU admission or death in the KCH sample associated with this score are shown in the figure below. All admissions presented with at least one of these risk factors (mean 4.4). 83% scored 3 or above (probability of admission to ICU or death = 13%), 65% scored 4 or above (probability = 21%).

Based upon these data, and the importance of radiographic severity, we selected a score of 3 if radiographic severity score is met, or 4 or higher otherwise as a threshold that captures patients with sufficient risk of progression to justify the risks of immune modulation. This accounted for 71% of the KCH sample. Specifically, individuals meeting this criterion had a 39% risk of admission to ICU or death, versus 9% in those that did not (odds ratio = 5.9).

The components of the score and the threshold may be adjusted as new clinical data become available.

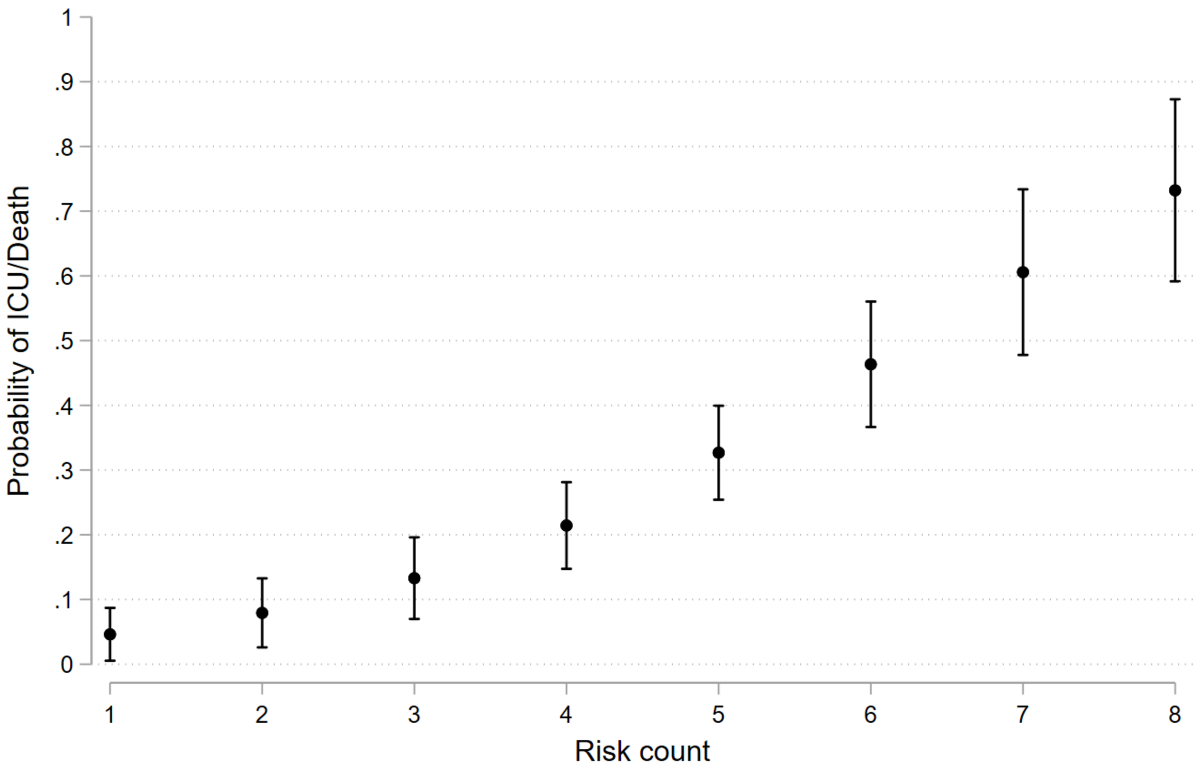

Figure 5. Risk of death or ICU admission. AUC for model = 0.75.

5.5 Study proposal

This study will assess the efficacy of immunomodulatory agents that target the dysregulated immune response that drives the severe lung, and other organ, damage. We will compare immunomodulation to standard-of-care. TACTIC-R will iterate an algorithm for use of clinical and biochemical phenotyping to:

a) Stratify patients to therapeutic arms according to probability of efficacy
b) Identify early indicators of failure of therapeutic strategy.

By collecting samples for genomics, transcriptomics, proteomics and immunological phenotyping, parallel studies associated with TACTIC-R will investigate host susceptibility factors for development of severe COVID-19-related disease and predictive biomarkers of response to therapeutic strategy.

The medications investigated for efficacy in this protocol are Baricitinib, and Ravulizumab. The anticipated sample size is 687 patients randomised in a 1:1:1 ratio across treatments with interim analysis at 375 patients.

TACTIC-R will use a platform design with interim analysis to make efficient decisions about efficacy and futility (e.g. lack of efficacy and risk of harm) of the trial treatments. The platform design concept is shown below. This enables us to stop recruiting to arms early where a clear decision can be made (as detailed in section 7.1). It also allows for the addition of further arms.

Platform design

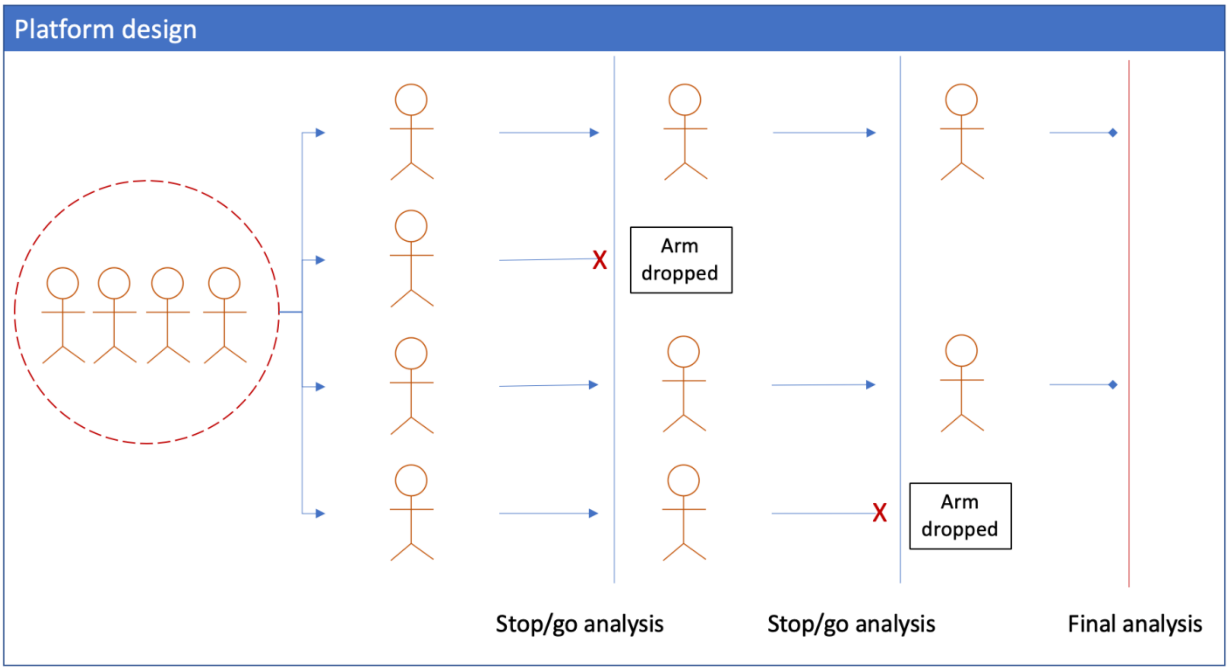

Figure 6. Platform design

5.6 Trial Hypotheses

We aim to test the hypotheses that:

a) Immune modulatory therapy is superior to standard of care alone;

b) Reduction of exaggerated host immune response to COVID-19 in patients at late stage 1/early stage 2 disease, reduces the composite of progression of these patients to organ failure or death and also reduces late sequelae of infection;

c) Clinical and biochemical markers can be used to stratify each patient to an effective therapeutic agent and can report early on efficacy of the therapeutic approach.

The design of the TACTIC-R trial incorporates serial interim analyses, at the discretion of the DMC, to identify:

a) Agents with efficacy, which can then be fast-tracked into NHS treatment pathways;

b) Agents with futility or significant adverse effects, which can then be removed from the study; and/or

c) New arms that can be added as potential beneficial drugs become available.

6 Information on Selected Treatments

6.1 Baricitinib

6.1.1 Mechanism of Action

Baricitinib is a selective and reversible inhibitor of Janus kinase (JAK)1 and JAK2. In isolated enzyme assays, baricitinib inhibited the activities of JAK1, JAK2, Tyrosine Kinase 2 and JAK3 with IC_{50} values of 5.9, 5.7, 53 and > 400 nM, respectively (data from published Baricitinib SmPC). There are a number of JAK/STAT inhibitors which are used in rheumatoid arthritis. A comparison of the inhibition of JAK1/3-, JAK2/2- and JAK1/2/TYK2-dependent cytokine pathways (McInnes et al 2019) indicates that Baricitinib exhibits slightly lower JAK1/3 inhibition (cytokines important in T cell and NK cell activation) than the comparators but all inhibit GM-CSF, G-CSF, IL6, IFNalpha. There is some IFNalpha inhibition, but baricitinib has lesser activity than tofacitinib for this anti-viral cytokine. (Table from McInnes et al 2019)

Baricitinib also decreases receptor-mediated endocytosis by inhibiting APR2-associated protein kinase 1 and also via G-associated kinase, and so is predicted to have anti-viral effects (not confirmed).

Table 1 IC_{50} values in CD4+ T cells, NK cells, and monocytes

Stimulation/pSTAT	CD4+ T cells			NK cells			Monocytes		
	Bari (nM)	Upa (nM)	Tofa (nM)	Bari (nM)	Upa (nM)	Tofa (nM)	Bari (nM)	Upa (nM)	Tofa (nM)
JAK1/3-dependent cytokines									
IL-2/pSTAT5	29	10**	11***	44	27*	15***	NS		
IL-4/pSTAT6	48	18***	18***	22	8*	8***	45	22**	35*
IL-15/pSTAT5	40	17***	15***	67	40*	22***	NS		
IL-21/pSTAT3	64	20***	22***	62	24**	21***	85	34	37
JAK2/2- or JAK2/TYK2-dependent cytokines									
IL-3/pSTAT5	NS			NS			26	12*	102***
G-CSF/pSTAT3	NS			NS			65	84	97**
GM-CSF/pSTAT5	NS			NS			30	13***	97***
JAK1/JAK2/TYK2-dependent cytokines									
IL-6/pSTAT3	61	58	56	NS			48	43	40
IL-10/pSTAT3	68	87	55	87	124	74	142	80***	104*
IFN-γ/pSTAT1	NS			NS			38	30	46***
IFN-α/pSTAT1	64	40	132***	76	69	121***	97	44**	163***
IFN-α/pSTAT3	27	17	51***	NS			14	6*	23***
IFN-α/pSTAT5	23	14	36**	NS			13	5**	22***

Table 1. IC_{50} values in CD4+ T cells, NK cells and monocytes

6.1.2 Rationale for use as Therapeutic in COVID-19-related disease

Baricitinib is administered orally once daily. It is licensed in the UK for treatment of rheumatoid arthritis, it is a relatively fast acting disease modifying anti-rheumatic drug and has the potential to be scaled up for use for a pandemic.

The JAK pathway is appealing as a therapeutic strategy in COVID-19 for three reasons:

First, the cytokine profile of patients transitioning from stage 2 to 3 is characterised by a striking elevation in IL-6.

IL-6 is a pleiotropic cytokine that stimulates diverse cellular responses such as proliferation, differentiation, survival, and apoptosis and can activate hepatocytes to release acute-phase proteins, including C-reactive protein (CRP). Elevated levels of IL-6 are found in the serum of patients with COVID-19 who progress to stage 3 disease and play a key role in both the pathologic inflammation and lung destruction which are hallmarks of the disease on intensive care. IL-6 is involved in diverse physiological processes such as migration and activation of T-cells, B-cells, and monocytes, leading to systemic inflammation. To achieve these downstream effects, IL-6 signals via the JAK1/2 receptor, activating the intracellular STAT pathway. Baricitinib potently blocks this pathway, and we believe that this can prevent the progression from stage 2 to stage 3 disease.

Administration of baricitinib results in a dose dependent inhibition of IL-6 induced STAT3 phosphorylation in whole blood from healthy subjects with maximal inhibition observed 2 hours after dosing which returned to near baseline by 24 hours. The efficacy and safety of baricitinib once daily was assessed in 4 Phase III randomised, double-blind, multicentre studies in patients with moderate to severe active rheumatoid arthritis. In patients with rheumatoid arthritis, decreases in serum C-reactive protein (CRP) were seen as early as 1 week after starting treatment and were maintained throughout dosing.

Second, baricitinib inhibits non-IL-6 pathways. Baricitinib inhibits Tyk2/Jak1 kinases, thereby targeting signals transduced through common gamma chain cytokine receptors (IL-2, IL-4, IL-7, IL-9, IL-15, and IL-21) as well as type I and II interferons. Although IL-6 is the most often cited driver cytokine in the published literature surrounding COVID-19, it is clear that the process is complex and driven by a complex network of inflammatory mediators. The broad array of cytokine pathways interrupted by baricitinib underpins its selection.

Third, the SARS-CoV2 virus enters cells via the ACE2 receptor. To gain entry, the receptor undergoes endocytosis. AP2-associated protein kinase 1 (AAK1) is a regular of endocytosis. Inhibition of endocytosis is a potential strategy for preventing viral entry into cells. Baricitinib is a high affinity AAK1 binding drug, thus potentially offering added anti-viral mechanisms of action.

Twelve consecutive adult patients admitted to an Italian hospital with COVID19-related disease were treated with Baricitinib 4mg od 2 weeks in an open-label, non-randomised study. The patients were also treated with lopinavir/ritonavir. One patient had a transaminitis at 10 days but there were no other adverse effects. All 12 patients treated with Baricitinib improved compared to (non-randomised) controls with respect to cough, dyspnoea, fever and oxygen saturations (Cantini et al 2020).

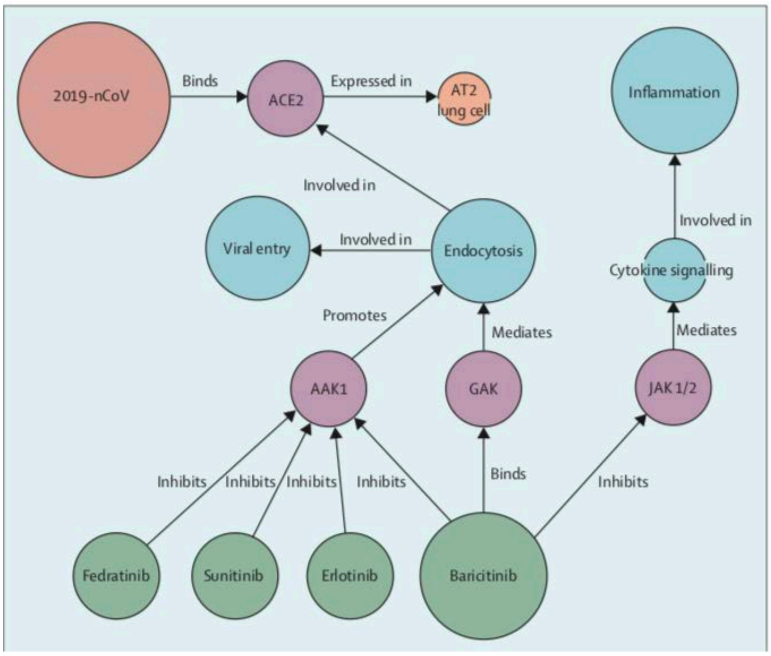

Figure 7. Rationale for Baricitinib

6.2 Ravulizumab

6.2.1 Mechanism of Action

Ravulizumab (Ultomiris, Alexion Pharmaceuticals) is a monoclonal antibody that binds to terminal complement protein C5 and prevents the complement-mediated destruction of cells. It is administered by intravenous infusion. Ravulizumab has a marketing authorisation in the UK for treating Paroxysmal Nocturnal Haemoglobinuria in adults.

6.2.2 Rationale for use as Therapeutic in COVID-19-related disease

The complement system consists of a series of soluble serine proteases, which circulate in the blood in inactive form. Activation of the complement cascade results in amplification of the inflammatory response and the formation of a membrane attack complex which perforates cell membranes, thereby causing cell death. The complement cascade can be activated by microbes, including viruses but the development of specific antibodies to a target, such as COVID19 spike protein, focusses complement activation, via the classical pathway, onto membranes expressing the target molecule; this would include infected cells. It would be expected that complement activation, in COVID-19-infected tissues would be markedly amplified after development of COVID-19-specific antibodies, i.e. in stage 2 of the disease. Since both complement components and antibodies circulate in the blood, it is plausible that complement-mediated attack contributes to tissue damage in sites other than the lung, including heart and kidney.

Data both from animal models of coronavirus infection and, recently, from histological analysis of skin and lung biopsy material from patients with COVID-19 provide evidence for complement cascade activation in the inflammatory second stage of disease (Gralinski et al 2018, Magro et al 2020). Since Ravulizumab inhibits activation of complement C5 (see Figure 3 above), it reduces or blocks effector pathways including complement-mediated cell killing and amplification of acute inflammation.

7 Trial Design

7.1 Statement of Design

Randomised, parallel arm, open-label Phase IV platform trial of immunomodulatory therapies in patients with late stage 1/stage 2 COVID-19-related disease, with a diagnosis based either on a positive assay or high suspicion of COVID-19 infection by clinical and radiological assessment.

We propose a platform trial design which will enable evaluation of multiple treatments simultaneously under a single protocol. This design would allow flexible features such as dropping treatments for futility, declaring one or more treatments superior to standard of care, or adding new treatments to be tested during the course of a trial. TACTIC-R is part of the TACTIC programme of research. This protocol will be aligned to an experimental medicine protocol (TACTIC-E) designed to test smaller numbers of COVID-19 infected patients with unlicensed medications. Standard of care data from TACTIC-R and TACTIC-E may be pooled within the programme.

Following confirmation of COVID-19 diagnosis and evidence for immune-activation, patients will be consented and then stratified to appropriate treatment arms, across which they will be randomised on a 1:1 basis.

7.1.1 Addition of treatment arms

The DMC can recommend the addition of new treatment arms. These will only be considered after it is clear that recruitment rate can support additional arms and after candidate(s) have been selected from the panel discussed by the TACTIC Drug Evaluation group, and subject to formal ethical and regulatory approvals. Subjects will then be enrolled into additional arms in a 1:1 ratio with the other active arms and the standard of care arm.

7.1.2 Termination of treatment arms

Subjects will continue to be enrolled in a treatment arm until one or more of the following have been demonstrated (as assessed by DMC on review of data collected):

1) Efficacy
2) Futility
3) Toxicity

If a treatment arm is discontinued due to lack of efficacy or safety concerns then all subjects currently being dosed will stop dosing of randomised IMP and no more subjects will be randomised to that arm.

If subjects have not received any study medication, then they can be re-randomised to one of the remaining arms.

Subjects in whom dosing has ceased will be considered to have completed active treatment, but will continue to be followed up as per the trial protocol until day 90.

Number of Participants
We plan to recruit between 687 (229 per arm) to 1167 (469 per arm) completed participants (modelled on a 3-arm design) across several UK centres. The full sample size calculation is described separately. The DMC will conduct interim analyses and will advise to stop an arm when either efficacy, or futility has been demonstrated, or if a significant safety signal is noted. Therefore each arm is likely to finish before reaching the maximum of 469 recruited.

7.2 Participants Trial Duration

The patient will remain in the study for up to 90 (+/- 7) days. The trial primary endpoint will be assessed at day 14. Follow up will continue for approximately 4 months to capture secondary endpoints.

7.3 Trial Objectives

7.3.1 Primary objective

- To determine if a specific immunomodulatory intervention reduces the composite of progression of patients with COVID-19-related disease to organ failure or death.

7.3.2 Secondary objectives

- To determine if a specific immunomodulatory intervention reduces severity of disease as assessed by the 7-point ordinal scale (see Figure 8);
- To determine if a specific immunomodulatory intervention reduces incidence of the individual endpoints of the composite
- To assess the safety and efficacy of the different treatment arms

	**Pulmonary 7-point scale**
**1**	Death
**2**	Invasive mechanical ventilation or ECMO
**3**	Non-invasive ventilation or high flow oxygen
**4**	Low flow oxygen
**5**	Hospitalised – no oxygen
**6**	Discharged; normal activities not resumed
**7**	Discharged; normal activities resumed

Figure 8. 7-point Ordinal Scale

7.3.3 Exploratory objectives

- To identify clinical or biochemical predictors of response to a trial intervention

7.4 Trial Outcome Measures

7.4.1 Primary outcome measure

- Time to incidence (up to and including Day 14) of any of the following events, whichever comes first:
    - Death
    - Invasive mechanical ventilation
    - ECMO
    - Cardiovascular organ support (balloon pump or inotropes)
    - Renal failure (estimated creatinine clearance (by Cockcroft-Gault formula) <15 ml /min), haemofiltration or dialysis.

7.4.2 Secondary outcome measures

- Change in clinical status as assessed on 7-point ordinal scale compared to baseline
- Time to each of the individual endpoints of the composite primary outcome measure
- Proportion of patients with adverse events of special interest in each treatment arm
- Time to $Sp0_2$ >94% on room air (excluding chronically hypoxic individuals)
- Time to first negative SARS-CoV2 PCR
- Duration of oxygen therapy (days)
- Duration of hospitalisation (days)
- All cause mortality at day 28
- Time to clinical improvement (defined as >2 point improvement from day 1 on 7-point ordinal scale)

7.4.3 Exploratory outcome measures

- Changes in biochemical predictors and immunoinflammatory signatures of therapeutic response and to inform algorithm development
- Clinical and biomarker predictors of therapeutic response

8 Selection and withdrawal of participants

8.1 Inclusion Criteria

To be included in the trial the participant must:

- be aged 18 and over
- have clinical picture strongly suggestive of COVID-19-related disease (with/without positive COVID-19 test) **AND**
    - Risk count (as defined above) >3

      OR
    - ≥3 if risk count includes “Radiographic severity score >3”
- be considered an appropriate subject for intervention with immunomodulatory in the opinion of the investigator
- be able to be maintained on venous thromboembolism prophylaxis or current maintenance therapy during inpatient dosing period, according to local guidelines

8.2 Exclusion Criteria

The presence of any of the following will preclude participant inclusion:

- Inability to supply direct informed consent from patient or from Next of Kin or Independent Healthcare Provider on behalf of patient
- Invasive mechanical ventilation at time of prior to dosing
- Contraindications to study drugs, including hypersensitivity to the active substances or any of the excipients
- Currently on any of the study investigational medicinal products
- Known unresolved *Neisseria meningitidis* infection
- Unwilling to be vaccinated against *Neisseria meningitidis* or receive prophylactic antibiotic cover until 2 weeks after vaccination
- Known active tuberculosis (no blood screening required)
- Known active Hepatitis B or C (no blood screening required); active varicella zoster.
- Concurrent participation in any interventional clinical trial including COVID-19-related disease trials (observational studies allowed)
- Patient moribund at presentation or screening
- Pregnancy at screening (or unwillingness to adhere to pregnancy advice in protocol)
- Unwillingness to adhere to breastfeeding advice in protocol.
- Either alanine transaminase or aspartate transaminase (ALT or AST) > 5 times the upper limit of normal
- Stage 4 severe chronic kidney disease or requiring dialysis (i.e. Cockcroft Gault estimated creatinine clearance < 30 ml /min)
- Currently receiving probenecid or chronic IVIG treatment
- Any medical history or clinically relevant abnormality that is deemed by the principal investigator and/or medical monitor to make the patient ineligible for inclusion because of a safety concern.

8.3 Treatment Assignment and Randomisation Number

Eligible patients will be randomised using a central web-based randomisation service called Sealed Envelope in a 1:1:1 ratio, stratified by site, to one of the following treatment arms (each in addition to standard of care (SoC)).

Arm 1: Baricitinib oral tablets (4mg OD) in addition to standard of care. In patients with a creatinine clearance between 30 and 60 ml/min inclusive or those aged ≥ 75, half dosing will be used.

Arm 2: Ravulizumab intravenous infusion (single dose, weight based dosing) in addition to standard of care

Arm 3: Standard of care

8.4 Treatment Cessation Criteria

- Alternative clinical diagnosis appears (i.e. no longer considered to have COVID-19-related disease)

- Progression to primary endpoint before dosing with any of the IMPs.
- SAR or SUSAR
- Confirmed **NEW** deep vein thrombosis or pulmonary embolism on imaging (if on Baricitinib arm)
- Withdrawal of patient consent

If the primary endpoint is reached then ongoing treatment with baricitinib may be discontinued at the discretion of the PI.

Patients who have been withdrawn from the trial treatment and are experiencing ongoing toxicity will be followed up until the adverse reaction comes to its conclusion. In the event of a patient being withdrawn from the trial treatment, they will continue to receive the most appropriate standard of care treatment available under the guidance of their treating clinician.

These treatment cessation patients will remain in the intention to treat analysis of the protocol and patients will continue to attend follow-up visits, when they are willing and able.

8.5 Consent withdrawal

Patients may withdraw their consent to participate in the trial at any time. No further trial procedures will be undertaken and no data or samples will be collected from the time of withdrawal.
However, data and samples collected up to the time of consent withdrawal will be included in the data reported for the trial. The Investigator should inform the coordination team as soon as possible and complete the consent withdrawal Case Report Form (CRF).

9 Trial Treatments

9.1 Treatment Summary

For the purpose of this trial the therapies in the treatment arms are all considered as Investigational Medicinal Products (IMP)s conducted with a Clinical Trial Authorisation. The 2 IMPs proposed are Baricitinib and Ravulizumab. All currently licensed drugs are being used outside of their licensed indications.

Further information regarding trial treatments is contained in the IMP manual.

9.1.1 Baricitinib

Baricitinib is a selective and reversible inhibitor of Janus kinase (JAK)1 and JAK2.

9.1.1.1 *Legal status*

Baricitinib is licensed for the treatment of moderate to severe active rheumatoid arthritis in adult patients who have responded inadequately to, or

who are intolerant to one or more disease-modifying anti-rheumatic drugs. Baricitinib may be used as monotherapy or in combination with methotrexate.

9.1.1.2 *Supply*

The IMP will be obtained from Eli Lilly and Company Limited. It will be distributed to authorised sites by a sponsor-appointed agent, with further details provided in the IMP manual. The initial supply will be sent to sites upon activation, with further supplies ordered by the site.

9.1.1.3 *Packing and Labelling*

Baricitinib is presented as a pink oblong film-coated tablet. Each tablet contains 2mg of baricitinib. Commercially available packs will be provided and labelling will comply with regulatory requirements.

9.1.1.4 *Storage conditions*

To be stored as per SmPC.

9.1.1.5 *Maximum duration of treatment of a participant*

Participants will receive the IMP as tablets once daily for 14 days. Treatment will be stopped at discharge or transfer to another healthcare facility (if classed as a different legal entity) regardless of the scheduled duration of therapy.

9.1.1.6 *Dose*

Participants will be given 4mg of baricitinib PO (2 x 2mg tablets, once daily) on days 1-14 PO.
See section 9.1.1.8 for dose adjustments for age and renal function.

9.1.1.7 *Administration*

The IMP is to be taken once daily with or without food at any time of the day. For patients unable to take tablets by mouth, these may be dispersed in water and administrated via a nasogastric tube.

9.1.1.8 *Dose modifications*

Patients with an estimated Cockcroft Gault creatinine clearance of 30-60 ml/min inclusive or patients ≥75 years at start of dosing will be given 2mg PO OD of baricitinib on days 1-14.

9.1.1.9 *Side effects*

Known reactions are summarised in section 4.8 of baricitinib SmPC approved by the MHRA for use in this trial.

9.1.1.10 *Management of toxicities associated with IMP*

Information concerning toxicity and management of toxicity is described in the SmPC.

When Baricitinib is prescribed for rheumatoid arthritis, blood monitoring for haematological, hepatic and metabolic adverse effects is arranged to start 2 weeks after initial dosing. Since the maximum treatment period with

Baricitinib in patients participating in TACTIC-R will be 14 days, the normal monitoring arrangements are inappropriate in the trial setting. However, participants in TACTIC-R will be inpatients who will have adverse effects monitored daily and will have blood tests, including full blood count and liver function tests performed serially through the treatment period. Daily reviews of results from TACTIC-R participants will be reviewed by site medical TACTIC investigators.

9.1.1.11 *Concomitant medications*

Details of drug-drug interactions are contained in the SmPC.

9.1.2 Ravulizumab

9.1.2.1 *Legal status*

Ravulizumab is licensed for the treatment of selected adult patients with paroxysmal nocturnal haemoglobinuria (PNH)

9.1.2.2 *Supply*

The IMP will be supplied by the marketing authorisation holder Alexion. It will be distributed to authorised sites by a sponsor-appointed agent with further details provided in the IMP manual. The initial supply will be sent to sites upon activation, with further supplies ordered by the site.

9.1.2.3 *Packing and Labelling*

Commercially available Ravulizumab (Ultomiris) will be supplied as 300mg concentrate for solution for infusion:
30 mL of sterile concentrate in a vial with a stopper and a seal.
Labelling will be in accordance with regulatory requirements.

9.1.2.4 *Storage conditions*

To be stored as per SmPC.

9.1.2.5 *Maximum duration of treatment of a participant*

Participants will receive the IMP as a single intravenous infusion.

9.1.2.6 *Dose*

Ravulizumab is administered intravenously at a concentration of 5mg/ml through a 0.2µm filter; it should not be administered as an intravenous push or bolus injection.

Ravulizumab weight-based dosing regimen

**Body weight range (kg)**	**Dose (mg)**
≥ 40 to < 60	2,400
≥ 60 to < 100	2,700
≥ 100	3,000

9.1.2.7 *Dose modifications*

No dose adjustment is required for patients aged 65 years or over, or for renal impairment. The safety and efficacy of ravulizumab have not been studied in patients with hepatic impairment; however pharmacokinetic data suggest that no dose adjustment is required in patients with hepatic impairment.

Dose modifications are not required for this trial.

9.1.2.8 *Side effects*

Known reactions are summarised in section 4.8 of ravulizumab SmPC approved by the MHRA for use in this trial.

9.1.2.9 *Management of Toxicities Associated with IMP*

Information concerning toxicity and management of toxicity is described in the SmPC.

Particular attention will be paid to the increased risk of bacterial meningitis with ravulizumab. Patients who have not been vaccinated against *Neisseria meningitides* with MenACWY and MenB during the last 5 years must receive appropriate antibiotic treatment until 2 weeks after vaccination (if not currently on antibiotics). Vaccinations should occur at approximately 28 days (if patient is stable) and should be tetravalent ACWY vaccine plus a serotype B vaccine. If vaccinations are not administered, antibiotic prophylaxis should continue for 8 months post treatment.

9.1.2.10 *Concomitant medications*

Details of drug-drug interactions are contained in the SmPC.

9.2 Active comparator products

Baricitinib + standard of care will be actively compared to standard of care alone. Ravulizumab + standard of care will be actively compared to standard of care alone. No comparisons will be made between active arms in this platform trial.

9.3 Accountability and dispensing

9.3.1 Dispensing

IMPs will be supplied against a prescription.

Refer to the IMP manual for details regarding ordering, stock levels, temperature monitoring and quarantine procedures.

9.3.2 Drug accountability

Accountability records detailing receipt, storage, dispensing and administration will be maintained by the participating site.

9.3.3 Returns and destruction

The nature of this study means that the return of IMPs dispensed to hospital wards may not be possible and returns are not, therefore, a formal sponsor requirement. As all IMPs are dosed as inpatients, evidence of dosing may be obtained from hospital administration records.

If returns are made, destruction according to local procedures is permitted, after successful local reconciliation and documentation. No further sponsor approval is required.

Refer to IMP manual for further details.

10 Procedures and assessments

Trial assessments and procedures will be performed by suitably qualified and delegated trial personnel as described in detail in the Trial Procedures Manual TPM.

10.1 Participant identification

Potential patients will be identified by an attending clinician upon arrival to the participating hospital if they are strongly suspected to be or are COVID-19 positive. Suitable patients will be approached and referred to the research team if appropriate. This may be achieved by reviewing inpatient medical notes (by a member of the clinical team or suitably qualified, delegated team member) or by discussion with clinical teams regarding their inpatients. Patients will be referred to the research team if they are interested in participating in this clinical trial.

There will be study advertisements placed in clinical areas, web-based (online/generic Trust emails/newsletters) and social media platforms. Research team members will monitor admissions, electronic track boards in the emergency department and admissions ward and may receive COVID result alerts to identify potential participants.

Once contact has been made with the patient, the research team will outline and explain the aims of the trial. A copy of the Patient Information Sheet will then be given to the patient who will have the opportunity to consider the information and discuss the trial with the trial staff and raise any queries before consenting to participate in the trial.

10.2 Consent

Informed consent should be obtained from each patient before enrolment into the study. In line with other urgent COVID -19 trials such as RECOVERY if the patient lacks capacity to give consent due to the severity of their medical condition (e.g. acute respiratory failure or delirium), then consent may be obtained from a relative

acting as the patient's legally designated representative. Further consent will then be sought with the patient if they recover sufficiently.

Due to the poor outcomes in COVID-19 patients who require ventilation (>50% mortality in one cohort), patients who lack capacity to consent due to severe disease (e.g. severe hypoxia), and for whom a relative to act as the legally designated representative is not immediately available, randomisation and consequent treatment will proceed with consent provided by a treating clinician (independent of the clinician seeking to enrol the patient) who will act as the legally designated representative. Consent will then be obtained from the patient's personal legally designated representative (or directly from the patient if they recover promptly) at the earliest opportunity.

10.3 Screening evaluation

10.3.1 Screening Assessments (Day -2 to Day -1)

Trial specific assessments will only be conducted after written informed consent has been provided. Due to the urgent nature of the study, it is anticipated that screening and baseline assessments will occur on the same day for most participants.

The results of screening tests must be available before randomisation and IMP dosing. Baseline tests will be done but results do not need to be available before dosing.

The screening/baseline visit will take place on inpatient wards/appropriate facilities in the hospital. The following procedures will be performed at this visit:

Screening procedures:

- Consent
- Review of medical history and examination and whole medical record
- Medication review
- Full blood count (including differential white cell count)*
- Calculated Cockcroft Gault creatinine clearance*
- ALT* or AST*
- CRP*
- Chest X-ray**
- Pregnancy test (blood)***
- Eligibility check of inclusion/exclusion criteria
- ECOG and MRC scores

*The results of these tests acquired up to 48hr before consent may be used to complete the screening and eligibility process.

**Clinically indicated chest X-ray will be used for radiology score. The TACTIC-R research protocol does NOT mandate a chest X-ray is done.

***Pregnancy test will not be done on post-menopausal women (for the purposes of this trial, postmenopausal is defined as being amenorrhoeic for greater than 2

years with appropriate clinical profile, e.g. age appropriate, history of vasomotor symptoms)

Eligible subjects should be randomised as soon as possible after eligibility is confirmed, with dosing occurring on the same day where possible.

10.3.2 Participant Randomisation

All patients screened for the trial will be assigned a unique participant ID number All screening tests must be available and checked by the delegated trial doctor before randomisation. The PI or delegate must sign the CRF to confirm eligibility after the screening process has been completed.

Suitable participants will be subsequently randomised at the participants' research site using a web-based online system. Randomisation notifications will be sent via email to research staff at the participating site including pharmacy as well as to the trial coordinator. Notifications will include information on drug allocation for the randomised patient. Further details can be found in the TPM.

10.4 Baseline Assessments (Day -2 to Day -1)

The following assessments will be undertaken either at the time of screening or soon after randomisation and the data points to be recorded at baseline are:

- Days since onset of symptoms
- Demographics and anthropomorphic data (age, height, weight etc)
- Vital signs (from medical records for e.g. heart rate, pulse, temperature etc as defined in TPM)
- Document current position on 7-point ordinal scale
- Obtain COVID-19 RTPCR result (if available)
- Extraction of clinical data from medical records (e.g. other bloods, radiology, etc.)
- Optional research blood samples and/or venous endothelial cell sampling (where units have capability)^
- ECOG and MRC scores

^Research blood samples at baseline are optional.

At selected sites, research blood samples may be taken for assays of blood biomarkers of response including but not confined to immunological and genomic transcriptomic and cellular analyses for future analysis. Venous endothelial cells may also be collected to determine the systemic vascular response to immune activation and treatment effects. This will be recorded in the CRF.

After randomisation all patients will receive baricitinib or ravulizumab according to the randomisation schedule, plus all ongoing standard of care treatment.

10.5 Trial assessments

Trial visits will be conducted at the trial sites.

10.5.1 Timing of in hospital assessments

Daily assessments will be done days 2-14 (or until discharge, whichever comes sooner):

- Vital signs (from medical records for e.g. heart rate, pulse, temperature etc as defined in TPM)
- Document current position on 7-point ordinal scale
- Obtain COVID-19 RTPCR results (if available)
- Review of adverse events

Additional assessments (Days 2, 6 and 14) ± 2 days

- Full blood count (including differential white cell count)*
- Calculated Cockcroft Gault creatinine clearance*
- ALT* or AST*
- Optional research blood sampling and/or venous endothelial cell sampling (where units have capability)
- Routine retrieval of clinical data

* The results of these tests acquired within a 96 hour window may be used.
The Day 14 or discharge assessments will be done where feasible.

10.5.2 Further assessments and end of trial visit

These visits (in person or by telephone consultation with participant or family member) will be held 28 (+/- 7 days) days and 90 days (+/- 7 days) after the first dosing visit.

Assessments will include the following:

- Discharge status
- Vaccination status (for ravulizumab arm only)
- Return to normal function status (numeric rating scale 0-10)
- Mortality status
- Adverse event reporting
- ECOG and MRC scores

At selected sites, research samples may be taken for assays of biomarkers of response including but not confined to immunological and genomic transcriptomic and cellular analyses for future analysis. Results of routine clinical investigations, such as echocardiograms, chest X-rays, CT thorax, lung function tests may be collected, if available.

Anonymised imaging for recruited patients may be retrieved from the medical record (during, before and after the index COVID-19 admission up to Day 90) and sent to a central imaging facility in the UK

10.6 Schedule of Assessments

Time and Events table for data collection during TACTIC-R Study

Data	Screening (Day -2 to Day -1)^	Baseline (Day-2 to Day -1)^	D1^	D2 *	D3	D4	D5	D6*	D7	D8	D9	D10	D11	D12	D13	Optional D14[@] or discharge date[@]	Follow up (~28days and 90 days)
Informed consent	x																
Eligibility criteria	x																
Medical history	x																
Physical examination	x																
Vital signs[#c]	x	x		x	x	x	x	x	x	x	x	x	x	x	x	x	
Height and weight[#c]	x																
Medication review	x			x				x								x	
Screening bloods (Full blood count including differential white cell count, creatinine, ALT or AST, CRP [#;] pregnancy test).	x [c]																
Routine retrieval of clinical data from the whole medical record[#]				x*				x*								x*	x*
Chest X-ray[#d]	x																x[e]
Days since onset of symptoms		x															
Demographics and anthropomorphic data		x															
7-point ordinal scale		x		x	x	x	x	x	x	x	x	x	x	x	x	x	
COVID-19 RTPCR (result may not be available prior to dosing[#]		x[#] (if not already taken)		x[#]	x[#]	x[#]	x[#]	x[#]	x[#]	x[#]	x[#]	x[#]	x[#]	x[#]	x[#]	x[#]	
Research blood and/or endothelial cell samjospling[a,b]		x[b]		x[b]				x[b]								x[b]	x[b]
Review of adverse events	x			x	x	x	x	x	x	x	x	x	x	x	x	x	x
Discharge status																	x
Vaccination status (ravulizumab arm only)																	x
Return to normal function status																	x
Mortality status																	x
ECOG and MRC scores	x	x															x
Baricitinib administration			x	x	x	x	x	x	x	x	x	x	x	x	x	x	
Ravulizumab administration			x														

[#]Results (e.g. haematology, biochemistry, immunology, microbiology, imaging) will be extracted from the patient record where available – no research specific test is required;

[a]Samples could be stored for assays of biomarkers of response including but not confined to immunological and genomic transcriptomic and cellular analyses for future analysis
[b] Research sampling is optional where units have capability – not mandatory
[c]The results of these tests acquired up to 48hr before consent may be used to complete the screening and eligibility process.
[d] Clinically indicated chest X-ray will be used for radiology score. The TACTIC-R research protocol does NOT mandate a chest X-ray is done.
[e] These assessments are optional depending on site resources.
*Can be done +/- 2 days
[@]Can be done +/- 2 days – this is not a mandatory assessment and will be done when feasible
^ Can be performed on the same day

10.7 End of Trial Participation

The patient's participation in the trial will end once they have completed their Day 90 follow-up visit or withdrawn their consent for the trial. However, all on-going ARs must continue to be followed after their participation has ceased until they have been resolved. Patients will return to normal standard of care when the treatment period has terminated, if the treatment arm they are on is terminated or on discharge.

10.8 Trial restrictions

Women of childbearing potential are required to use adequate contraception for the duration of the trial (and for 8 months after if randomised to the Ravulizumab arm only) upon completion of the last treatment. This includes:

- Intrauterine Device (IUD)
- Hormonal based contraception (pill, contraceptive injection or implant etc)
- Barrier contraception (condom and occlusive cap e.g. diaphragm or cervical cap with spermicide)
- True abstinence (where this is in accordance with the participants' preferred and usual lifestyle)
- Sterilisation (e.g. tubal ligation, oophorectomy, hysterectomy, vasectomy)

Men are required to use adequate contraception for the entire duration of the trial (and for 8 months after if randomised to the Ravulizumab arm only) upon completion of the last treatment. This includes:

- Barrier contraception (condom and spermicide) even if female partner(s) are using another method of contraception or are already pregnant (also to protect male partners from exposure to the trial IMPs etc)
- True abstinence (where this is in accordance with the participants' preferred and usual lifestyle)

11 Assessment of Safety

11.1 Definitions

11.1.1 Adverse event (AE)

Any untoward medical occurrence in a participant or clinical trial participant administered a medicinal product and which does not necessarily have a causal relationship with this treatment.
An adverse event can therefore be any unfavourable and unintended sign (including an abnormal laboratory finding), symptom, or disease temporally associated with the use of an investigational medicinal product, whether or not considered related to the investigational medicinal product.

Please note: The Sponsor expects that all adverse events are recorded in the medical notes from the point of Informed Consent regardless of whether a participant has yet received a medicinal product. ARs, AESI, SAE and SARs should be recorded in the relevant pages in the CRFs. SUSARs should be reported as per Section 11.6.

11.1.2 Adverse reaction to an investigational medicinal product (AR)

All untoward and unintended responses to an investigational medicinal product related to any dose administered. All adverse events judged by either the reporting investigator or the sponsor as having a reasonable causal relationship to a medicinal product qualify as adverse reactions. The expression reasonable causal relationship means to convey in general that there is evidence or argument to suggest a causal relationship.

11.1.3 Unexpected adverse reaction

An adverse reaction, the nature, or severity of which is not consistent with the applicable reference safety information (RSI) When the outcome of the adverse reaction is not consistent with the applicable RSI this adverse reaction should be considered as unexpected.

The term “severe” is often used to describe the intensity (severity) of a specific event. This is not the same as “serious,” which is based on participant /event outcome or action criteria.

11.1.4 Serious adverse event or serious adverse reaction (SAE / SAR)

Any untoward medical occurrence that at any dose:

- results in death
- is life-threatening
- requires hospitalisation or prolongation of existing inpatients´ hospitalisation
- results in persistent or significant disability or incapacity
- is a congenital anomaly or birth defect.
- is an important medical event - Some medical events may jeopardise the participant or may require an intervention to prevent one of the above characteristics/ consequences. Such events (hereinafter referred to as ‘important medical events’) should also be considered as ‘serious’

Life-threatening in the definition of a serious adverse event or serious adverse reaction refers to an event in which the participant was at risk of death at the time of event; it does not refer to an event which hypothetically might have caused death if it were more severe.

11.1.5 Suspected Unexpected Serious Adverse Reaction (SUSAR)

A serious adverse reaction, the nature and severity of which is not consistent with the information set out in the Reference Safety Information.

11.1.6 Reference Safety Information (RSI)

A list of medical events that defines which reactions are expected for the IMP within a given trial and thus determining which Serious Adverse Reactions (SARs) require expedited reporting.

The RSI is contained in a clearly identified section of the Summary of Product Characteristics (SmPC) or the Investigator’s Brochure (IB).

For this trial the Reference Safety Information is:
Section 4.8 of the SmPC for:

Olumiant (Baricitinib), dated 22 November 2019 and Ultomiris (Ravulizumab), dated 02 July 2019 approved by the MHRA for use in this trial.

11.2 Expected Adverse Reactions/Serious Adverse Reactions (AR /SARs)

All expected Adverse Reactions are listed in the latest MHRA approved version of the RSI as specified in section 11.1.6. This must be used when making a determination as to the expectedness of the adverse reaction. If the adverse reaction meets the criteria for seriousness, this must be reported as per section 11.5.

11.3 Expected Adverse Events/Serious Adverse Events (AE/SAE)

The following adverse events are known manifestations of COVID-19 related disease. They will not be recorded in the medical notes as part of this COVID-19 intervention trial. If these events are considered serious they will be recorded in the trial CRF:

- Chills
- Cough
- Bloody sputum
- Sore throat
- Nasal symptoms: blocked or runny nose
- Ear pain
- Wheezing
- Chest tightness
- Shortness of breath
- Muscle aches
- Joint pain
- Fatigue
- Headache
- Confusion
- Loss of appetite
- Abdominal pain
- Nausea/vomiting
- Diarrhoea
- Sore, red, gritty eyes
- Skin rash

11.4 Evaluation of adverse events

The Sponsor expects that adverse events (apart from expected AEs) are recorded in the medical notes from the point of Informed Consent regardless of whether a participant has yet received a medicinal product. Individual adverse events should be evaluated by the investigator. This includes the evaluation of its seriousness, and any relationship between the investigational medicinal product(s) and/or concomitant therapy and the adverse event (causality).

11.4.1 Assessment of seriousness

Seriousness is assessed against the criteria in section 11.1.4. This defines whether the event is an adverse event, serious adverse event or a serious adverse reaction

11.4.2 Assessment of causality

Definitely: A causal relationship is clinically/biologically certain. **This is therefore an Adverse Reaction**

Probable: A causal relationship is clinically / biologically highly plausible and there is a plausible time sequence between onset of the AE and administration of the investigational medicinal product and there is a reasonable response on withdrawal. **This is therefore an Adverse Reaction.**

Possible: A causal relationship is clinically / biologically plausible and there is a plausible time sequence between onset of the AE and administration of the investigational medicinal product. **This is therefore an Adverse Reaction.**

Unlikely: A causal relation is improbable and another documented cause of the AE is most plausible. **This is therefore an Adverse Event.**

Unrelated: A causal relationship can be definitely excluded and another documented cause of the AE is most plausible. **This is therefore an Adverse Event.**

Unlikely and Unrelated causalities are considered NOT to be trial drug related
Definitely, Probable and Possible causalities are considered to be trial drug related

A pre-existing condition must not be recorded as an AE or reported as an SAE unless the condition worsens during the trial and meets the criteria for reporting or recording in the appropriate section of the CRF as specified in section 11.1.1 and 11.3.

11.4.3 Clinical assessment of severity

Mild: The participant is aware of the event or symptom, but the event or symptom is easily tolerated

Moderate: The participant experiences sufficient discomfort to interfere with or reduce his or her usual level of activity

Severe: Significant impairment of functioning; the subject is unable to carry out usual activities and / or the participant's life is at risk from the event.

11.4.4 Recording of adverse events

Adverse events (apart from expected AEs) will be recorded in the medical notes only. Due to the underlying clinical condition of the trial population it is not practicable to report all adverse events in this trial and it is thought that excessive safety reporting may detract from the main objectives of the trial. Rather, only AEs of special interest (AESI) should be recorded and reported as detailed in section 11.8.
Adverse reactions should be recorded in the medical notes and the appropriate of the CRF and/or AR log. Serious Adverse Reactions should be reported to the sponsor as detailed in section 11.5.

11.5 Recording and Reporting SAEs and SARs

All serious adverse events will be recorded (except where the protocol stated otherwise – see section 11.3) in the trial data collection tools. All will be reported SAEs to the Chief Investigator using the trial specific SAE form within 24 hours of their awareness of the event

The Chief Investigator is responsible for ensuring that the assessment of all SAEs for relatedness and expectedness is completed and the onward notification of all SARs Sponsor immediately but not more than 24 hours of first notification. The sponsor has to keep detailed records of all SARs reported to them by the trial team. All SAEs/SARs may be reported by the trial team to Alexion Pharmaceuticals UK and Eli Lilly and Company UK in line with the contractual requirements.

The Chief Investigator is also responsible for prompt reporting of all Serious Adverse Reactions to the competent authority (e.g. MHRA) of each concerned Member State if they could:

- adversely affect the health of participants
- impact on the conduct of the trial
- alter the risk to benefit ratio of the trial
- alter the competent authority's authorisation to continue the trial in accordance with Directive 2001/20/EC

The completed SAE/SAR form must be emailed. Details of where to report the SAE/SARs can be found on the 'TACTIC-R' SAE/SAR form and the front cover of the protocol.

11.6 Reporting of Suspected Unexpected Serious Adverse Reactions (SUSARs)

All suspected adverse reactions related to an investigational medicinal product (the tested IMP and comparators) which occur in the TACTIC-R trial, and that are both unexpected and serious (SUSARs) are subject to expedited reporting. Please see section 11.1.6 for the Reference Safety Information to be used in this trial.

11.6.1 Who should report and whom to report to?

The Sponsor delegates the responsibility of notification of SUSARs to the Chief Investigator. The Chief Investigator must report all the relevant safety information previously described, to the:

- Sponsor
- Competent authorities in the concerned member states (eg MHRA)
- Ethics Committee in the concerned member states

The Chief Investigator shall inform all investigators concerned of relevant information about SUSARs that could adversely affect the safety of participants. SUSARS may also be reported to the license holder if requested.

11.6.2 When to report?

11.6.2.1 Fatal or life-threatening SUSARs

The CI must inform the Sponsor of any fatal SUSAR immediately but within 24 hours of the site investigator awareness of the event. The MHRA and Ethics Committee must be notified as soon as possible but no later than **7 calendar days** after the trial team and Sponsor has first knowledge of the minimum criteria for expedited reporting.
In each case relevant follow-up information should be sought and a report completed as soon as possible. It should be communicated to all parties within an additional **8 calendar days**.

11.6.2.2 Non-fatal and non-life-threatening SUSARs

All other SUSARs and safety issues must be reported to the Sponsor immediately but within 24 hours of the site investigator awareness of the event. The MHRA and Ethics Committee must be notified as soon as possible but no later than **15 calendar days** after the trial team and Sponsor has first knowledge of the minimum criteria for expedited reporting. Further relevant follow-up information should be given as soon as possible.

11.6.3 How to report?

11.6.3.1 Minimum criteria for initial expedited reporting of SUSARs

Information on the final description and evaluation of an adverse reaction report may not be available within the required time frames for reporting. For regulatory purposes, initial expedited reports should be submitted within the time limits as soon as the minimum following criteria are met:
a) a suspected investigational medicinal product
b) an identifiable participant (e.g. trial participant code number)
c) an adverse event assessed as serious and unexpected, and for which there is a reasonable suspected causal relationship
d) an identifiable reporting source

and, when available and applicable:

- an unique clinical trial identification (EudraCT number or in case of non-European Community trials the sponsor's trial protocol code number)
- an unique case identification (i.e. sponsor's case identification number)

11.6.3.2 Follow-up reports of SUSARs

In case of incomplete information at the time of initial reporting, all the appropriate information for an adequate analysis of causality should be actively sought from the reporter or other available sources. Further available relevant information should be reported as follow-up reports.
In certain cases, it may be appropriate to conduct follow-up of the long-term outcome of a particular reaction.

11.6.3.3 Format of the SUSARs reports

Electronic reporting is the expected method for expedited reporting of SUSARs to the competent authority. The format and content as defined by the competent authority should be adhered to.

11.7 Pregnancy Reporting

All pregnancies within the trial (either the trial participant or the participant's partner) should be reported to the Chief Investigator and the Sponsor using the relevant Pregnancy Reporting Form within 24 hours of notification. Pregnancies must be reported for all patients for the duration of their trial participation until 8 months after treatment completion.

All pregnancies will be reported by the trial team to Alexion Pharmaceuticals UK in line with the contractual requirements

Pregnancy is not considered an AE unless a negative or consequential outcome is recorded for the mother or child/fetus. If the outcome meets the serious criteria, this would be considered an SAE.

11.8 Adverse events of special interest (AESIs)

For this trial, the following adverse events will be recorded as AESIs:

- Venous thromboembolism
- New infections requiring antimicrobials

Each Principal Investigator must report all AESIs to the CI using the AESI form in a timely manner. If the AESI is deemed to be serious, then the reporting procedure for an SAE should be followed as detailed in section 11.5,

12 Toxicity – Emergency Procedures

In the event of an acute hypersensitivity to any of the IMPs, supportive care will be given to the patient according to local clinical procedures.

13 Evaluation of Results (Definitions and response/evaluation of outcome measures)

Please refer to section 7.5 Trial Outcome Measures

13.1 Response criteria

Please refer to section 7.5 Trial Outcome Measures

14 Storage and Analysis of Samples

Research blood samples that are to be analysed at a later stage are to be stored in an appropriate manner at the local site. Only the research team will have access to these samples and analyse when logistics permit. The samples will be labelled with the patient trial-specific identifiers

15 Statistics

15.1 Statistical methods

The primary endpoint will compare the experimental treatments to control using Cox proportional hazards, adjusting for important baseline prognostic predictors (age, gender, ethnicity, radiological severity score, underlying health condition,

neutrophils, CRP, and recruiting site). Estimates, 95% confidence intervals and p-values will be provided for the treatment effects on the hazard ratio (HR) scale. The main analyses will be conducted by the trial statistician following the intention-to-treat principle.

Secondary endpoints will be analysed using a similar regression methodology, as suitable for the nature of the endpoint (binary, categorical, continuous, time-to-event).

15.2 Interim analyses

Interim analyses are scheduled at the end of period 1 (125 per arm) or 4 weeks after first patient is enrolled whichever is sooner (period 1). At the interim analysis a decision to progress to period 2 will be made by the DMC based on data relating to the primary outcome and safety data. This decision will be informed by Bayesian posterior distributions for the treatment effects on the primary outcome of each experimental treatment. Specifically, the DMC will be provided with estimates of the probabilities for each treatment arm relative to control relating to efficacy (HR<1), moderate or greater efficacy (HR<0.80), similarity (0.80<HR<1.25), and harm (HR>1).

The subsequent timing of any future interim analyses will be determined by the DMC.

The DMC will decide which arms will be continued for recruitment. The standard care arm will always be continued. At least one or more experimental arms will be continued into period 2 giving a possible 3 choices. If deemed necessary, only one experimental arm will be continued into period 3 (see section 15.3).

To inform the choice, Bayesian posterior distributions will be inferred for the treatment effects of each experimental treatment, assuming vague priors. These will be used to calculate, for each of the possible combination of arms to continue, predictive posterior probabilities that a future trial with a fixed total number of participants will result in any statistically significant positive results using a conventional 2.5% 1-sided hypothesis test: a Bayesian equivalent to predictive power. The total number of patients used for these calculations will be varied for consideration by the DMC, in the light of recruitment rates, but a reference case will be a two armed trial comparing a treatment to standard of care, with a total N=229. The choice that provides the highest probability of ultimately achieving a significant result may be the recommendation, but the totality of information including external information will be considered by the DMC. An arm may be stopped early for efficacy if Pocock's bounds are exceeded (p<0.001). An arm may be stopped early for futility, outside of the predictive posterior probability framework, if the posterior probability of a negative treatment effect exceeds 80%, or a clear safety signal is observed.

The DMC will be presented with data relating to the primary outcome, the ordinal clinical status measure, and safety data. If the results are deemed inconclusive (e.g. there is a clear signal for efficacy but a potential signal for harm), the DMC may recommend progressing to period 3.

15.3 Number of Participants to be enrolled

There is not a fixed sample size for this study and the only predetermined aspect is to perform an interim analysis after 125 patients are recruited to each arm, 375 in total assuming 3 arms. Thereafter the DMC has the ability to recommend which arms are to continue or allow additional arms, and for how many subsequent patients to recruit before the next interim analysis. Provisionally, there will be a second interim after 229 patients per arm, and potentially then a third interim after 469 per arm, to be agreed or modified with the DMC at each preceding interim analysis.

If the trial does not continue after the first interim analysis, the sample size will be 375. Using our provisional estimates, where only one experimental treatment is continued the sample size will be 583, and where both experimental arms are continued the sample size will be 687. Although we do not anticipate progressing beyond this second interim analysis, if at that stage the findings are inconclusive with respect to efficacy or harm, and the DMC considers it necessary and it is logistically feasible, a maximum of one experimental arm may continue into period 3. This would mean recruiting 469 patients in total to both the experimental arm and control arm; resulting in a maximum sample size of 1167.

If additional experimental arms are added, these will have an initial sample size of 125 and will then progress to the provisional sample size of 229 dependent on DMC recommendation.

The provisional target sample size of 229 per arm is based on a reference study with a fixed sample size involving two arms comparing treatment to standard of care, which is similar to other trials in this population. Specifically, 229 per arm is based on 80% power to detect a moderate clinically relevant difference in the primary outcome (one-sided alpha=.025), and also a key secondary outcome, namely the ordinal clinical status at 14 days. The use Pocock's bounds for stopping for efficacy does not require adjustment for multiple testing.

i) The target sample size will allow for a detection of a 40% lower hazard (HR=.6) of the composite endpoint involving the first observed of invasive mechanical ventilation, renal replacement therapy, inotropic support, balloon pump, or death. This assumes the event rate in the control group is 20% at 14 days, which is consistent with the numbers requiring invasive mechanical ventilation or dying for the first 200 admission with COVID-19 at King's College Hospital. The expected event rate in the treatment group is approximately 12%.

ii) The target sample size will allow for a detection of 60% increased odds of better clinical status at 14 days on the 7-point COVID-19 ordinal outcomes scale. Based on data from the first 200 admission with COVID-19 at King's College Hospital, this represents an expected difference in the number scoring either of the two highest points on the scale, representing discharge, of 55.0% in the control group and 66.5% in the experimental group, and an expected difference in the number scoring the

lowest point on the scale, death, of 15% in the control group and 9.8% in the experimental group.

The effects for each period before the interim analysis are given below, where we assume 125 patients per arm are recruited by 4 weeks, and the subsequent periods are four weeks each.

	Estimated Number of Patients per arm	Hazard Ratio, 80% power (Composite of events)	Odds ratio, 80% power (ordinal outcome scale)
Period 1	125	0.5	1.9
Period 2	229 (further 104)	0.6	1.6
Period 3	469 (further 240)	0.7	1.4

15.4 Procedure to account for missing or spurious data

Given data are collected mainly in hospital, with key outcome data being time to a fixed event (e.g. ICU admission or death), we anticipate the level of missing data to be negligible. Where missing data relates to an event, we will assume the event has not occurred up to the last known data point available for the patient, with the data censored at that point and not included in the further analyses. A similar approach will be undertaken for longitudinal mixed models under the assumption that data are missing at random. Only patients with at least one data point post-randomisation will be included in the analyses.

15.5 Definition of the end of the trial

The end of trial will be the date 18 months after the last patient's last visit to allow sufficient time to complete all primary, secondary, and exploratory endpoints and their corresponding analyses, and if applicable, all re-analyses of samples.

16 Data handling and record keeping

16.1 CRF

All data will be transferred into a Case Report Form (CRF) which will be labelled using a participant's unique trial ID and partial date of birth. All trial data in the CRF must be extracted from and be consistent with the relevant source documents. The CRFs must be completed, dated and signed by the investigator or designee in a timely manner. It remains the responsibility of the investigator for the timing, completeness, legibility and accuracy of the CRF pages. The CRF will be accessible to trial coordinators, data managers, the investigators, Clinical Trial Monitors, Auditors and Inspectors as required.

The investigator will retain the original of each completed CRF page at site. Completed CRFs should be emailed to the trial coordination centre. The investigator will also supply the trial coordination centre with any required, anonymised background information from the medical records as required.
A trial specific data management plan will describe in detail the data management processes using the CRF and the trial database.

Any trial related documentation that is sent to the trial coordination centre must not contain patient identifiable data.

All CRF pages must be clear, legible and completed in ink. Any errors should be crossed with a single stroke so that the original entry can still be seen. Corrections should be inserted and the change dated and initialled by the investigator or designee. If it is not clear why the change has been made, an explanation should be written next to the change. Typing correction fluid must not be used.

16.2 Source Data

To enable peer review, monitoring, audit and/or inspection the investigator must agree to keep records of all participating participants (sufficient information to link records e.g., CRFs, hospital records and samples), all original signed informed consent forms and copies of the CRF pages.

Source data may include but is not limited to:

- Signed informed consent forms
- Patient medical records (electronic or paper)
- Blood and imaging results (electronic or paper)
- Prescriptions
- Sample logs
- Clinical research forms/folders

16.3 Data Protection & Participant Confidentiality

All investigators and trial site staff involved in this trial must comply with the requirements of the the General Data Protection Regulation 2018, Data Protection Act 2018 and Trust Policy with regards to the collection, storage, processing, transfer and disclosure of personal information and will uphold the Act’s core principles.

17 Data Monitoring Committee

The DMC will be comprised of an independent group, as defined in a separate charter document which will define the role of the DMC. The charter document will be generated prior to opening the trial.

The DMC will be responsible for the review of all safety and efficacy (but not exploratory) as detailed below.

Review of data at Interim Analysis 1, 2 and 3 to determine:

a) If there is clear evidence of efficacy in any arm; in this case the DMC may recommend that the data are published and the agent provided in the care pathway for CRC.
b) If there is a safety signal in any arm; in this case, the DMC may recommend termination of the relevant arm.

18 Trial Steering Committee

The TACTIC-R TSC committee is a multidisciplinary group consisting of a group of member who jointly have responsibility for the design, conduct and evaluation of the clinical research project.

The role of the TSC is to provide overall supervision for a trial on behalf of the Trial Sponsor and Trial Funder and to ensure that the trial is conducted to the rigorous standards set out in the Department of Health's Research Governance Framework for Health and Social Care and the Guidelines for Good Clinical Practice. Further description of the TSC role in this trial is specified in a separate charter.

19 Ethical & Regulatory considerations

19.1 Ethical committee review

Before the start of the trial or implementation of any amendment we will obtain approval of the trial protocol, protocol amendments, informed consent forms and other relevant documents e.g., advertisements and GP information letters if applicable from the REC. All correspondence with the REC and HRA will be retained in the Trial Master File/Investigator Site File.

Annual reports will be submitted to the REC in accordance with national requirements. It is the Chief Investigator's responsibility to produce the annual reports as required.

19.2 Regulatory Compliance

The trial will not commence until a Clinical Trial Authorisation (CTA) is obtained from the MHRA. The protocol and trial conduct will comply with the Medicines for Human Use (Clinical Trials) Regulations 2004 and any relevant amendments.

Development Safety Update Reports (DSURs) will be submitted to the MHRA in accordance with national requirements. It is the Chief Investigators responsibility to produce the annual reports as required.

19.3 Protocol Amendments

Protocol amendments must be reviewed and agreement received from the Sponsor for all proposed amendments prior to submission to the HRA, REC and/or MHRA.

The only circumstance in which an amendment may be initiated prior to HRA, REC and/or MHRA approval is where the change is necessary to eliminate apparent, immediate risks to the participants (Urgent Safety Measures). In this case, accrual of new participants will be halted until the HRA, REC and/or MHRA approval has been obtained.

19.4 Peer Review

The TACTIC-R protocol has been reviewed by the TACTIC consortium as well as the Musculoskeletal and Respiratory Translational Research Collaboration (TRC) groups.

19.5 Declaration of Helsinki and Good Clinical Practice

The trial will be performed in accordance with the spirit and the letter of the declaration of Helsinki, the conditions and principles of Good Clinical Practice, the protocol and applicable local regulatory requirements and laws.

19.6 GCP Training

All trial staff must hold evidence of appropriate GCP training or undergo GCP training prior to undertaking any responsibilities on this trial. This training should be updated every 2 years or in accordance with your Trust's policy.

20 Sponsorship, Financial and Insurance

The trial is sponsored by Cambridge University Hospitals NHS Foundation Trust. The trial will be funded by Eli Lilly and Company UK Ltd., Alexion Pharmaceuticals UK.

Cambridge University Hospitals NHS Foundation Trust, as a member of the NHS Clinical Negligence Scheme for Trusts, will accept full financial liability for harm caused to participants in the clinical trial caused through the negligence of its employees and honorary contract holders. There are no specific arrangements for compensation should a participant be harmed through participation in the trial, but no-one has acted negligently.

21 Monitoring, Audit & Inspection

The investigator must make all trial documentation and related records available should an MHRA Inspection occur. Should a monitoring visit or audit be requested, the investigator must make the trial documentation and source data available to the Sponsor's representative. All participant data must be handled and treated confidentially.

The Sponsor's monitoring frequency will be determined by an initial risk assessment performed prior to the start of the trial. A detailed monitoring plan will be generated detailing the frequency and scope of the monitoring for the trial. Throughout the course of the trial, the risk assessment will be reviewed and the monitoring frequency adjusted as necessary.

Remote monitoring will be conducted for all participating sites. The scope and frequency of the monitoring will be determined by the risk assessment and detailed in the Monitoring Plan for the trial.

22 Protocol Compliance and Breaches of GCP

Prospective, planned deviations or waivers to the protocol are not allowed under the UK regulations on Clinical Trials and must not be used.

Protocol deviations, non-compliances, or breaches are departures from the approved protocol. They can happen at any time, but are not planned. They must be adequately documented on the relevant forms and reported to the Chief Investigator and Sponsor immediately.

Deviations from the protocol which are found to occur repeatedly will not be accepted and will require immediate action and could potentially be classified as a serious breach.

Any potential/suspected serious breaches of GCP must be reported immediately to the Sponsor without any delay.

23 Publications policy

Ownership of the data arising from this trial resides with the trial team and the sponsor. On completion of the trial the data will be analysed and tabulated and a Final Trial Report prepared. However, given the nature of this international pandemic, preliminary data may be reported prior to the completion the study, or if interim analyses are adequate for dissemination of critical safety or efficacy data. Any bloods done as part of the protocol or as part of observational studies will need to adhere to the pre-agreed publications policy of the TACTIC core research trial team. The sponsor will provide, if practicable, advanced notice of any publications to Alexion Pharma UK. At conclusion of the study a fully anonymised dataset will be placed in the public domain. Data sharing within a federated consortium of UK investigators across the four nations will be adopted.

24 References

- Ai et al 2020. Correlation of Chest CT and RT=PCR Testing on Coronavirus Disease 2019 (COVID-19) in China: A Report of 1014 Cases. Radiology; DOI: 10.1148/radiol.2020200642
- Al-Omari et al 2018 MERS coronavirus outbreak: Implications for emerging viral infections. Diagnostic Microbiol and Infect Disease 93: 265-285; doi.org/10.1016/j.diagmicrobio.2018.10.011Guan et al 2020. Clinical Characteristics of Coronavirus Diseae 2019 in China. NEJM DOI: 10.1056/NEJMoa2002032
- Cantini et al. Baricitinib therapy in COVID-19: A pilot study on safety and clinical impact. J Infect. (2020) doi: 10.1016/j.jinf.2020.04.017
- Gao et al 2020 Highly pathogenic coronavirus N protein aggravates lung injury by MASP-2-mediated complement over-activation. *medRxiv* https://doi.org/10.1101/2020.03.29.20041962
- Gautret et al 2020. Clinical and microbiological effect of a combination of hydroxychloroquine and azithromycin in 80 COVID-19 patients with at least a six-day follow up: an observational study – published ahead of press by Lover, A. A. Quantifying treatment effects of hydroxychloroquine and azithromycin for COVID-19: a secondary analysis of an open label non-randomized clinical trial. *medRxiv* 2020.03.22.20040949 (2020) doi:10.1101/2020.03.22.20040949.
- Gralinski and Baric 2015. Molecular pathology of emerging coronavirus infections. J Pathol 2015: 235: 185-195; DOI: 10.1002/path.4454
- Gralinski et al 2018. Complement activation contributes to severe acute respiratory syndrome coronavirus pathogenesis. Am Soc Microbiol 2018; 9: e01753 https://mbio.asm.org/content/9/5/e01753-18
- Guan et al 2020. Clinical Characteristics of Coronavirus Diseae 2019 in China. NEJM DOI: 10.1056/NEJMoa2002032
- Huang C, Wang Y, Li X, Ren L, Zhao J, Hu Y, et al. Clinical features of patients infected with 2019 novel coronavirus in Wuhan, China. Lancet. 2020;395(10223):497–506.

- Kanne et al 2020 Essentials for Radiologists on COVID-19: An Update – Radiology Scientific Expert Panel.
  Radiology doi.org/10.1148/radiol.2020200527
- Liu, Y. *et al.* Clinical and biochemical indexes from 2019-nCoV infected patients linked to viral loads and lung injury. *Sci. China Life Sci.* **63**, 364–374 (2020).
- Maharaj R King's Critical Care Evidence Summary 9/3/20 (King's College Hospital)
- McInnes et al 2019 Comparison of baricitinib, upadacitinib ad tofacitinib mediated regulation of cytokine signalling in human leukocyte subpopulations. Arthritis Res Ther. 2019; 21: 183.
- Mehta et al 2020 Covid-19: consider cytokine storm syndromes and immunosuppression; The Lancet https://doi.org/10.1016/S0140-6736(20)30628-0
- Merle et al 2015. Complement system part II: role in immunity. Front Immunol 2015: https://doi.org/10.3389/fimmu.2015.00257
- Peiris et al 2003. Clinical progression and viral load in a community outbreak of coronavirus-associated SARS pneumonia: a prospective study. Lancet 361: 1767-72
- Ramos-Casals et al Adult Haemophagocytic Syndrome 2014 The Lancet doi.org/10.1016/ S0140-6736(13)61048-X
- Richardson et al 2020 Baricitinib as potential treatment for 2019-nCoV acute respiratory disease. The Lancet https://doi.org/10.1016/ S0140-6736(20)30304-4
- Ruan, Q., Yang, K., Wang, W., Jiang, L. & Song, J. Clinical predictors of mortality due to COVID-19 based on an analysis of data of 150 patients from Wuhan, China. *Intensive Care Med.* (2020) doi:10.1007/s00134-020-05991-x.
- Russell, C. D., Millar, J. E. & Baillie, J. K. Clinical evidence does not support corticosteroid treatment for 2019-nCoV lung injury. *The Lancet* **395**, 473–475 (2020).
- De Wilde et al 2011 Cyclosporin A inhibits the replication of diverse coronaviruses J Gen Virol 2011 92: 2542
- Wang, D. *et al.* Clinical Characteristics of 138 Hospitalized Patients With 2019 Novel Coronavirus–Infected Pneumonia in Wuhan, China. *JAMA* **323**, 1061–1069 (2020).
- Xie, J. *et al.* Critical care crisis and some recommendations during the COVID-19 epidemic in China. *Intensive Care Med.* 1–4 (2020) doi:10.1007/s00134-020-05979-7.
- Zhou et al 2020 Utility of Ferritin, Procalcitonin and C-reactive protein in Severe Patients with 2019 Novel Coronavirus Disease. 10.21203/rs.3.rs-18079/v1

Safety Reporting Flow Chart

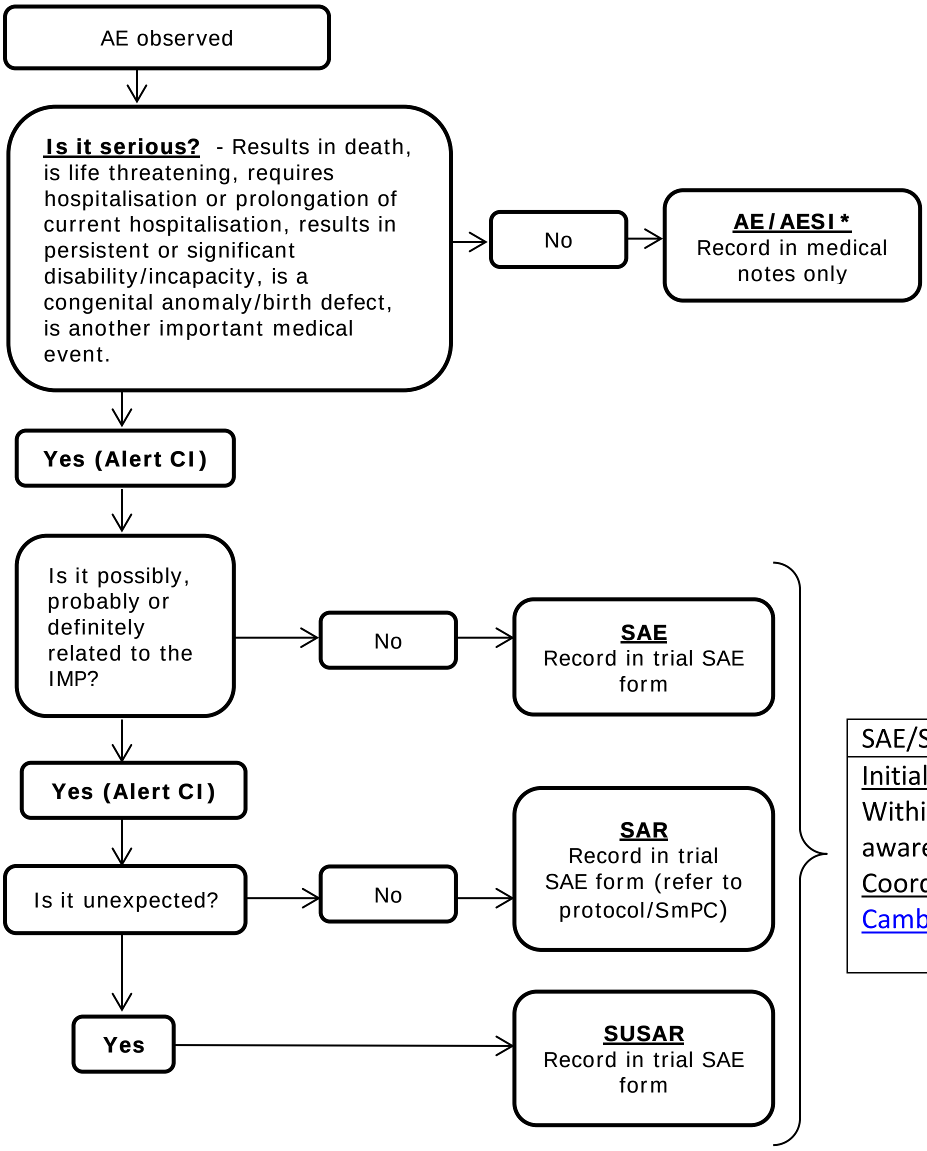

SAE/SAR/SUSAR reporting
Initial report<br>Within 24 hours of investigator awareness report to:<br>Coordinator:<br>Cambs.cardiovascular@nhs.net

SUSAR expedited reporting
The Chief Investigator must report a SUSAR to the Sponsor, REC and MHRA within statutory timelines. Each SUSAR requires the entry of relevant data and information by the Chief Investigator into the eSUSAR reporting system. A copy of this report should be provided to the CCTU PV team, collating these on behalf of the sponsor.

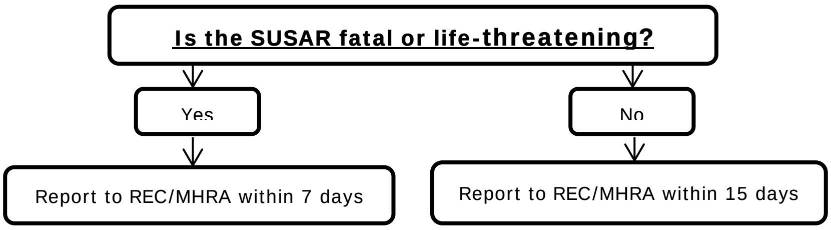

*AESI will also be recorded in the AESI form
